# The impacts of tidal wetland loss and coastal development on storm surge damages to people and property: a Hurricane Ike case-study

**DOI:** 10.1038/s41598-023-31409-x

**Published:** 2023-03-21

**Authors:** Zaid Al-Attabi, Yicheng Xu, Georgette Tso, Siddharth Narayan

**Affiliations:** 1 Department of Coastal Studies, Integrated Coastal Programs, East Carolina University, Greenville, NC USA; 2 Department of Marine Sciences, Marine Sciences Center, University of Basrah, Basrah, Iraq; 3 Integrated Coastal Sciences PhD Program, East Carolina University, Greenville, NC USA

**Keywords:** Natural hazards, Environmental impact

## Abstract

Coastal wetlands protect communities during hurricanes by reducing storm surge flooding and damages. Previous studies have quantified surge reduction benefits of wetlands, but there is less understanding of how the combination of wetland loss and coastal development influences the spatial distribution of flood extents and damages. In this study we integrate a high-resolution 2-D hydrodynamic model with land-use/land-cover change analyses to assess the effects of total wetland loss, decadal wetland loss, and coastal development on storm surge damages in Galveston Bay, Texas. We measure storm surge flood extents from Hurricane Ike for three scenarios: (i) 2008 Baseline; (ii) 2008 No Wetlands, and (iii) 2019 “Present-day H. Ike”. We find that during Hurricane Ike in 2008, the total loss of coastal wetlands would have increased damages by a net ~ USD $934 million or 12.8% of baseline damages. For the 2019 Present-day H. Ike scenario, we found very few wetlands were lost between 2008 and 2019. If Hurricane Ike had occurred in 2019, damages would have been higher by ~ $2.52 billion or 34.6%, almost entirely due to increased real estate value and new coastal development. Our findings suggest that, while increase in economic exposure is a key driver of storm surge risks in Galveston Bay, effective wetland conservation continues to reduce these risks.

## Introduction

Hurricane-generated storm surges have resulted in major economic losses and fatalities in the United States over the last century. Between 1900 and 2017, Hurricanes in the continental U.S. resulted in $2 trillion in economic damages as well as more than 16,000 deaths^1,2^. The risk of storm surge flooding can be generally characterized by the Hazard – Exposure – Vulnerability framework^3^. Here, Hazard refers to the storm surge flood extent driven by high water levels at the shoreline, Exposure refers to the number of assets or people flooded, and Vulnerability is the degree to which these assets and people are affected by storm surge flooding.

Total water levels at the shoreline that result in inland flooding during a storm are principally driven by a combination of storm surge and tide, with contributions from other ocean circulation conditions, like, for example, offshore currents^4^. Storm surges are often the dominant cause of damages during a hurricane. For instance, inland flooding due to storm surge was the biggest component of damage during Hurricanes Katrina in 2014^5^, Ike in 2008^6^, Sandy in 2014^7^ and most recently, Hurricane Ian in 2022^8^. The U.S. East and Gulf Coasts are regions with high exposure and vulnerability to flooding hazards from storm surges, aggravated by sea-level rise. Storm surge hazards in these generally flat floodplains can extend several miles inland from the coastal area depending on the topography, hurricane intensity, and tidal water levels during the surge.

While storm surge is a key driver of flood risks, coastal development also influences risks by increasing exposure to these hazards^9,10^. The combined effects of climate change and coastal development are projected to significantly increase future hurricane damage as a proportion of U.S. economic growth^11^. Coastal development, by driving exposure, can increase flood risks rapidly and over short periods of time. GIS-based assessments of sea-level rise and population projections indicate that, across the U.S., a sea-level increase of 0.9 m can put more than 4 million people at risk of flooding by 2100^12^. Multiple studies have investigated the influence of coastal development on exposure to sea-level rise and storm surge hazards and associated socio-economic damages^11,13^. Most of these studies focus on projected coastal development and associated increases in exposure to risk within the floodplain [e.g. Ref.^14^]. Coastal development, however, not only increases exposure to storm surge risk but can further aggravate this risk by removing natural inter-tidal wetlands that have been shown to reduce storm surge flood hazards^15^.

Inter-tidal wetlands are an important natural barrier that can mitigate exposure and vulnerability to storm surges by reducing flood extents and heights hazards and thus avoiding hurricane damages and deaths^16–18^. A number of studies have evaluated the physics of surge attenuation across wetlands and the subsequent benefits of wetlands to reduce storm surge^19^ and property damages^20–23^. Sheng et al.^19^ found that without vegetation cover, some parts of Miami would have experienced catastrophic flooding during Category 5 Hurricane Andrew in 1992^19^. Narayan et al.^20^ showed that tidal salt marsh wetlands avoided flood damages of ~ $625 million across several states along the northeast of the U.S. Atlantic coast during Hurricane Sandy in 2018, out of a total of $60 billion in damages, and found a strong positive correlation between wetland extent and flood damage reduction^20^. Sheng et al.^23^ found that these wetland benefits varied widely by location, between 8 and 52% of storm surge-related loss avoided, during Hurricane Sandy and other hypothetical storm events^23^. In Galveston Bay, Highfield et al.^24^, using statistical models, found that marsh wetland extent significantly influenced flood insurance claims, together with distance from the coast^24^. Though this study did not explore the spatial distribution of these effects, they highlight the importance of considering the presence of landscape features including barrier islands as these are intrinsically linked to the distribution of storm surge flood extents and to the effects of wetlands on these flood extents.

In this manuscript, we provide a first exploration of wetland loss and coastal development as two inter-related aspects that influence the spatial distribution of coastal flood risk during storm surges. As wetlands are lost to sea-level rise and coastal development, their ability to protect coasts against storm surges diminishes^9^. Our manuscript examines how changes to land-cover and land-use within the floodplain, including wetland loss, drive the spatial distribution of storm surge flood risk. We explore the effect of these changes on flood risk using process-based models with grid resolutions that are able to capture the influence of landscape features including barrier islands and major navigation channels such as the Intra-Coastal Waterway. By combining storm surge models with spatial land-cover and land-use analyses our manuscript provides a first evaluation of how total wetland loss, decadal wetland loss and coastal development together influence storm surge flood damages in a highly populated, hurricane-prone region.

Galveston Bay is a densely populated, hurricane-prone region that has sustained major flooding and destruction of coastal property during past storms including Hurricane Ike in 2008 and Hurricane Harvey in 2017. Galveston Bay is also home to large extents of wetlands, despite intensive human development across the shoreline and the presence of a major shipping channel^25–27^. A few previous studies have investigated the individual effects of coastal development and wetlands on flooding in Galveston Bay using empirical and statistical models at significantly lower resolutions relative to our study, and to date we know of none that have examined the combined effect of these factors on storm surge risk. Guannel et al.^28^ used an empirical, static 1-D model applied over multiple shore-perpendicular transects^28^ to assess the influence of sea-level rise on the coastal protection value of coastal wetlands, and Brody et al.^29^ explored the effect of palustrine wetland loss on rainfall-based flood risks using spatial statistical methods^29^, though neither of these studies examined the influence of development on risk. A multidisciplinary project carried out by Harte Research Institute investigated the social and economic impact of sea level rise on Harris, Brazoria, Galveston, Chambers, and Liberty counties that surround Galveston Bay^30^, though this study did not evaluate the effects of wetlands or coastal development on storm surge damage. Using Galveston Bay as a test-case, our study presents a novel, high resolution, spatially explicit assessment of the combined impacts of tidal wetlands and coastal development on storm surge risks to people and property. In 2008, Hurricane Ike made landfall in Galveston as a Category 2 Hurricane and became one of the most costly and damaging storms in U.S. history^31^. Although Ike was only a category 2 storm when it made landfall, it had a large wind field which created a large storm surge that caused significant flooding and major destruction of coastal property, especially on the eastern side of Galveston Bay. Total structural and property damages from Hurricane Ike across the Caribbean countries and United States are estimated at more than $29.5 billion^32^ with an estimated loss of life of 112 people directly and indirectly related to the storm along the U.S Gulf Coast regions^33,34^. Areas to the right side of the eyewall of Hurricane Ike, that were exposed to extreme onshore hurricane winds, generally recorded higher storm surge levels with the maximum surge level of 5.3 m recorded as far as 29 km inland in Chambers County, Texas^32^.

To assess the impacts of tidal wetland loss and coastal development on storm surge damages, we integrate Delft3D, a 2-D hydrodynamic storm surge model, with spatial land-cover and land-use change analyses using multiple publicly available datasets between the years 2008 and 2019 and for three comparative scenarios. First, we setup and validate a high resolution –5.56 km in deep ocean to 86 m in coastal and nearshore regions—Delft3D model to simulate storm surge flood extents and heights during Hurricane Ike in the Galveston Bay region. We then assess storm surge flood extents from Hurricane Ike for three scenarios: (1) 2008 Baseline with 2008 mean sea-level, wetland and land-cover data; (2) 2008 No Wetlands with 2008 mean-sea level and land-cover but assuming all coastal wetlands are lost to open water; and (3) 2019 “Present-day Hurricane Ike” with 2019 mean-sea level including sea-level rise since 2008, and 2019 wetland and land-cover. Flood heights are assessed only for storm surge and not for waves. We integrate storm surge flood heights from these three scenarios with publicly available spatial datasets of population, land-cover, land-use, and economic value to assess storm surge damages. We use public land use and property tax data for 2019 to assess total economic values in the flooded area. For 2008, we derive economic values based on available datasets on land-cover and land-use for 2008 and 2019. To assess economic damages, we use depth-damage functions to calculate, in a spatially explicit manner, the expected damages to the economic value of residential, commercial, and agricultural land-use types due to the flooding for each scenario. Damage values from all scenarios are compared to understand how the distribution of wetlands influenced storm surge damages during H. Ike in 2008 and how new coastal development and wetland loss in the subsequent decade have influenced these risks. All dollar amounts are presented as 2019 dollars.

## Results

### Hurricane Ike flooding and damages in 2008

**Figure 1:**
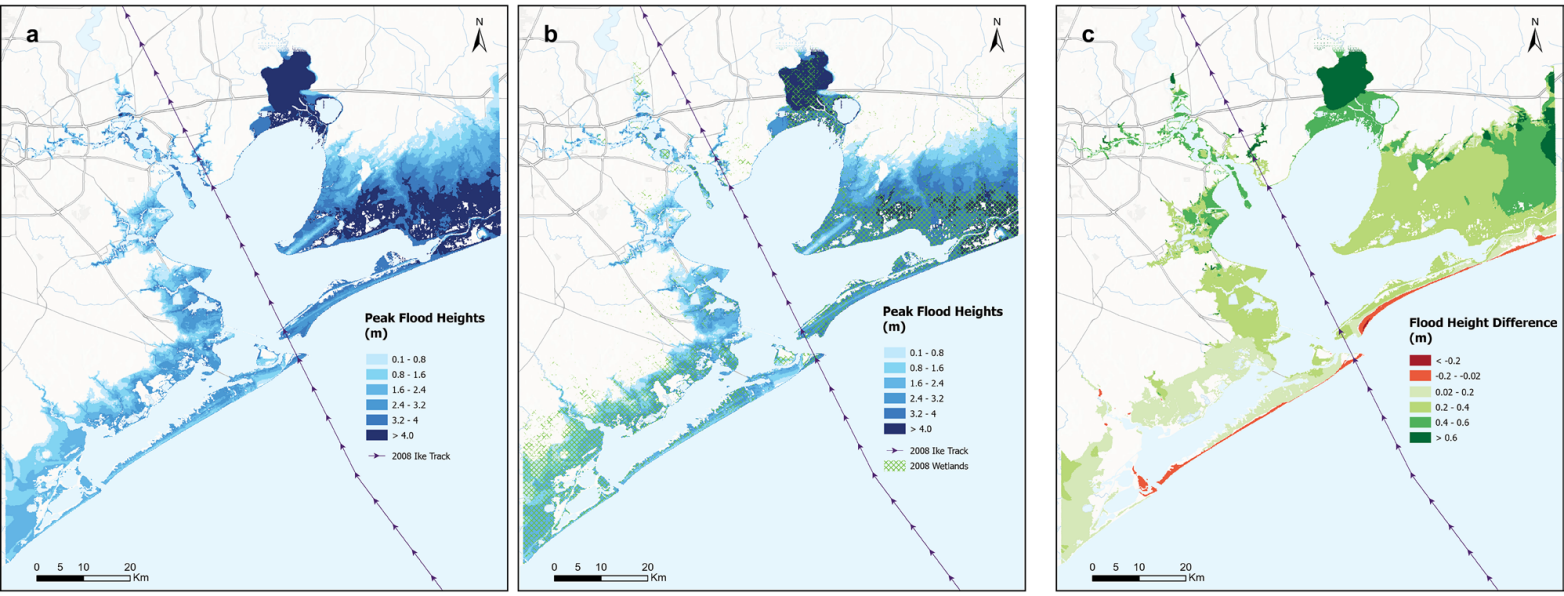
Simulated peak flood heights (m) showing the difference between flood elevations and the topographical (i.e. ground) surface from Hurricane Ike over regions in Galveston Bay for: (**a**) 2008 No wetlands, (**b**) 2008 Baseline, and (**c**) Difference (No Wetlands 2008 – Baseline 2008). Topographical and flood elevations are all relative to MSL.

The storm surge from Hurricane Ike flooded 55% of the floodplain (Table 1). We estimate that the storm surge affected 143,598 people, or ~35% of the population in the region and caused ~ $7.27 billion in storm surge damages. For both 2008 damage scenarios, higher flood peaks occur mostly in the eastern part of Galveston Bay (3–4.8 m) (Fig. 1a,b). This is due to landward hurricane winds that pushed water on to coastal areas. Lower flood peak values are seen in the western side of the bay (< 2 m), where the left side of Hurricane Ike was mostly located, with winds blowing seaward. Hurricane Ike also flooded other parts of coastal Texas and neighboring states (Fig. S6). The storm surge model performs well against observed tidal gauge water levels and high water marks, with a maximum root mean square error of 0.14 m for tidal gauge observations and 0.68 m for the high water marks (Table S1; Figs. S14, S15).

**Table 1:** Total wetland area, total flooded area and total storm surge damages from Hurricane Ike for each county in the study area for all three scenarios (2008 Baseline, 2008 No Wetland and 2019 “Present-day Hurricane Ike”).

County	Total wetland area (km^2^)		Total flooded area** (km^2^)			Total storm surge damage** by scenario (in 2019 $US) x $1 million		
	2008	2019	2008 Baseline	2008 no wetland	2019 “present-day H. Ike”	2008 baseline	2008 no wetland	2019 “present-day Hurricane Ike”
Galveston	198	186	468	483	467	5463	5878	7274
Harris	20	18	102	124	101	1335	1787	1899
Chambers	357	351	805	876	805	220	279	276
Brazoria*	249	238	284	288	283	251	259	336
Liberty	4	4	17	18	18	–	–	–
Total	828	797	1676	1789	1674	7270	8204	9787

All dollar values rounded up to the nearest 1,000,000.

*Only the part of the county flooded by Hurricane Ike is included in this analysis.

**Only where flood height is above 0.1 m.

### Wetland influence on storm surge flooding and damages during Hurricane Ike in 2008

Inter-tidal wetlands caused a notable reduction in flood elevation by 0.25–0.9 m (Fig. 1c) in most regions surrounding Galveston Bay during Hurricane Ike. These wetlands reduced the total peak flood extent by 112 km^2^ during Hurricane Ike in 2008 (Fig. 1c) and protected more than 18,000 people from this flooding. Within the study area of 3000 km^2^, the total size of wetlands cover (Emergent Herbaceous wetlands) in 2008 was 828 km^2^, and the total population was 405,730 people.

**Figure 2:**
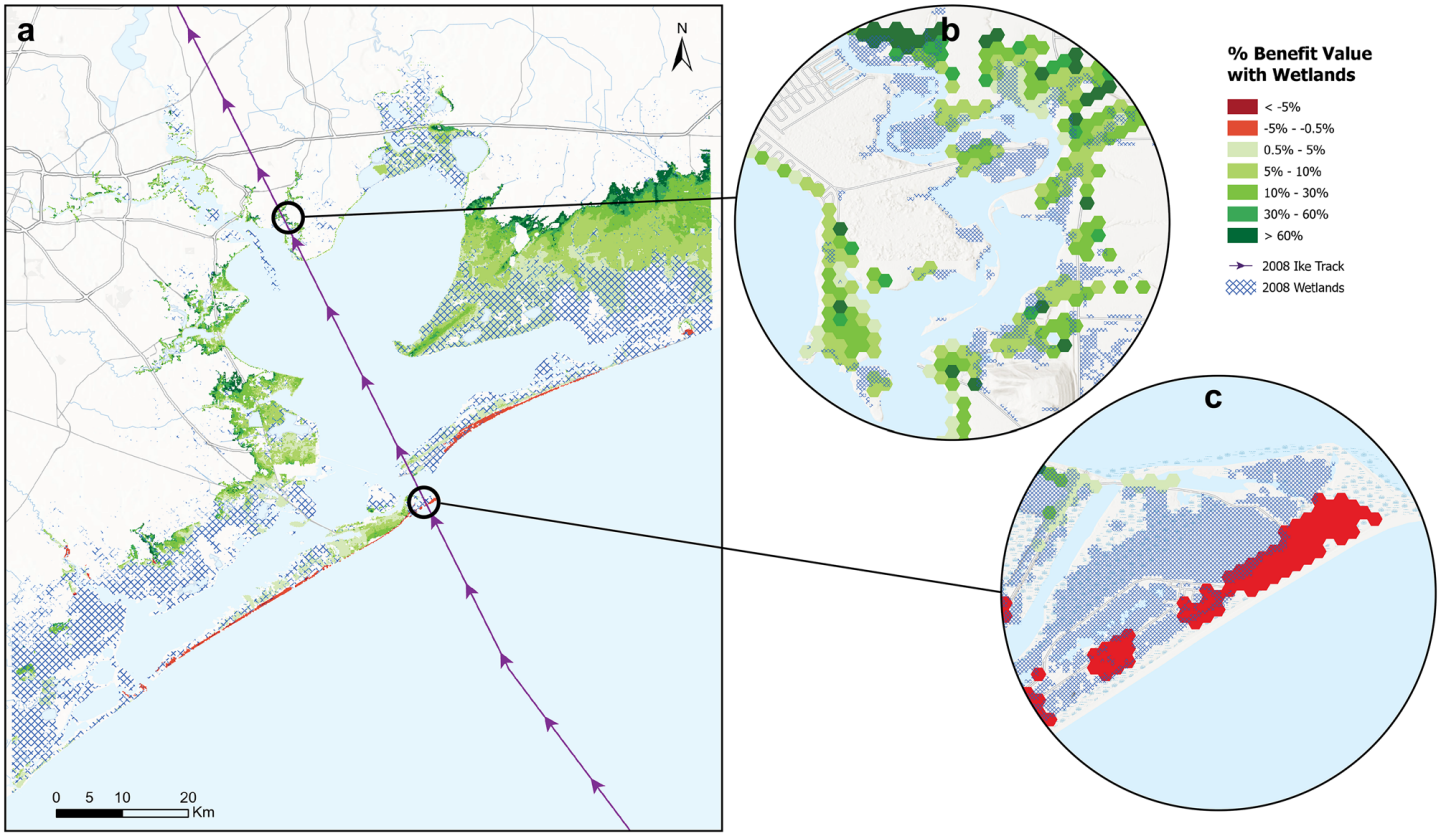
(**a**) Map shows the percentage benefit value of wetlands cover from 2008, calculated as the ratio of the difference in damages: (2008 No wetlands-2008 Baseline)*100/2008 Baseline. Inset panels (**b**) and (**c**) show detailed examples of positive and negative percentage benefit values, respectively, along H. Ike’s track for Houston Metropolitan and Galveston Island coastlines.

The reduction in flood extents due to the presence of wetlands translated to a net reduction in economic flood damages of ~ US$934 million or 12.8% of baseline losses. Among the flooded regions, Galveston and Harris counties benefited the most from the presence of wetlands with more than $400 million in flood damages avoided in each county, with coastal regions in the Houston Metropolitan area receiving significant benefits (Table 1; Fig. 2a,b).

Our study estimates the average value of wetlands in the Galveston Bay region for storm surge damage avoidance as $1.1 million per km^2^ during Hurricane Ike, though unit wetland benefits vary widely by location. During Hurricane Ike, Galveston County received an estimated ~ $415 million in storm surge reduction benefits from 198 km^2^ of coastal wetlands, translating to an economic benefit of ~ $2.1 million per km^2^ of wetlands in this county, whereas in Brazoria county, where losses and wetland extents were lower overall, wetlands provided benefits of ~ $32,000 per km^2^ (Table 1).

Total wetland loss would have resulted in 60% of the floodplain and 40% of the population (162,069 people) being flooded due to the storm surge, i.e. an additional 18,471 people compared to the baseline. Total wetland loss would have increased by more than double, the total area that experienced an extreme storm surge of > 4 m, and would have increased the number of people who experienced a > 4 m surge by ~ 25% (Supplementary Table S4). We however find that wetlands benefits for avoiding storm surge damage are greater at lower peak flood heights, with more than $780 million, i.e. more than 80% of the total wetland benefits, being received when peak flood heights were lower than 2.5 feet (Supplementary Fig. S13).

Interestingly we also find that wetlands increase peak flood depths and flood damages in some locations, by ~ $20 million, or 0.28% of total baseline losses). This negative effect of wetland cover is seen mostly in the outer-lying coastal regions of the barrier islands, for properties that are located seaward of the wetlands (Fig. 2c).

### Impact of coastal development and wetland change on storm surge damages for a 2019 “present-day Hurricane Ike”

**Figure 3:**
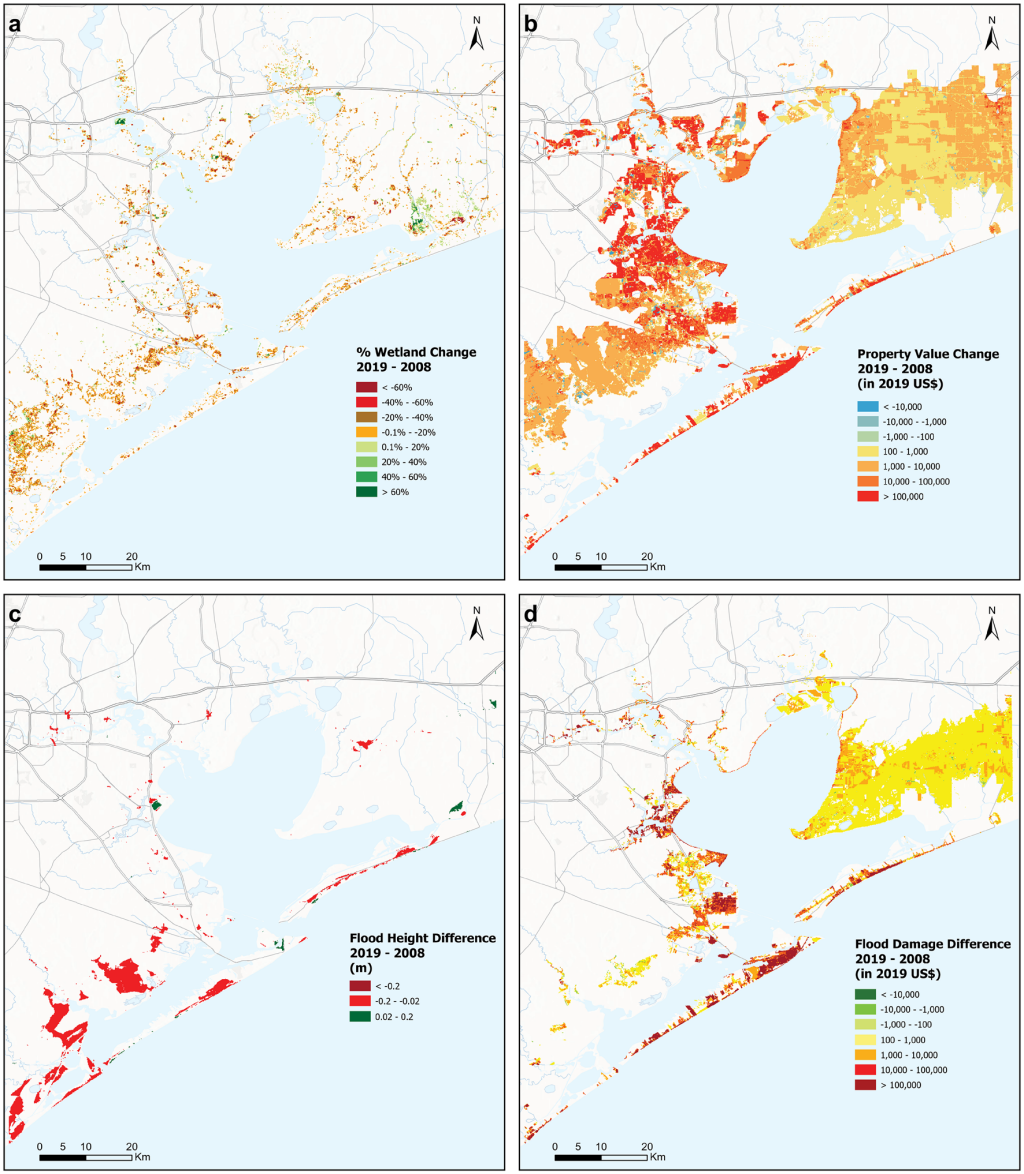
(**a**) Change in wetland cover between 2008 and 2019 in Galveston Bay: orange and green areas denote loss and gain in wetland cover, respectively, and light-yellow areas denote wetlands that did not change. (**b**) Property value change between 2008 and 2019, including increases in real estate prices in red, decreases in blue and no change in yellow (**c**) Difference in peak flood heights (i.e. difference between flood elevations and topographical elevations with respect to the MSL datum) between with 2019 “Present-Day Hurricane Ike” and 2008 Baseline scenarios, (**d**) Difference in economic storm surge damages between 2019 “Present-Day Hurricane Ike” and 2008 Baseline scenarios.

Our study shows that if Hurricane Ike had re-occurred in 2019, total storm surge damages would have been ~ 9.79 billion. This represents an increase in damages from 2008 of ~ 2.52 billion or 34.6%. We find that increases in economic value, in the form of new coastal development and increases in real estate values, are responsible for 99.7% of the total increase in economic damages from storm surge over the last decade (Fig. 3a–c). Almost 90% of this increase in risk due to economic activity is driven by the increase in real estate values within the floodplain between 2008 and 2019.

Increase in damages only due to new development, without considering the increase in real estate values, is ~ US$ 269 million, or ~ 11% of the total increase in damages. A comparison of land-cover and land-use data between 2008 Baseline and 2019 “Present-Day Hurricane Ike” scenarios shows that approximately 3% of the floodplain experienced land cover change between 2008 and 2019, most of this as urban development (Fig. 3a,d). The area of developed land increased by 52 km^2^ (8%) between 2019 and 2008, all of which was flooded. The largest increases in risk for new development were across industrial ($114 million), residential ($84 million) and commercial ($60 million) lands.

The study found that new coastal development is also associated with a very small amount of wetland loss that further aggravates this risk. Net wetland loss over this decade was approximately 31 km^2^ (3.7% of 2008 wetland extent), with some areas experiencing a loss and other areas experiencing a net gain in wetland extents (Fig. 3a) resulting in a net increase in storm surge damages of ~ $7 million, which translates to 0.27% of the total increase in damages, and 2.6% of the increase in damages due to new development alone. Gross wetland loss was ~ 15.95 km^2^ (1.9% of 2008 wetland extent), most of it due to the replacement of wetlands with infrastructure (Fig. 3a,b). Some parts of the floodplain also gained wetlands over this decade, to a total of 6.68 km^2^ of wetland area (0.8% of 2008 wetland extent).

## Discussion

This study presents a novel assessment of the combined impact of coastal development and wetland loss drivers on the total amount and spatial distribution of storm surge flooding and damages across a decadal timescale relevant for flood risk management and decision-making. While it is widely recognized that these drivers individually influence risks by modifying hazards and exposure, our study presents a first exploration of the combined impact of wetland loss and coastal development in terms of economic flood damages from storm surges. The study also highlights the extent to which changes in real estate value drive increases in overall risk.

Storm surge flooding was responsible for a large portion of the total damages from Hurricane Ike. While it is difficult to accurately validate our damage estimates, our estimate of $7.27 billion in storm surge damages slightly underestimates total damage estimates for Hurricane Ike^32,35–37^. Alvarez and Plocheck^37^ estimate that Hurricane Ike resulted in total damages of $15.6 billion, $8.9 billion of which was from storm surge in Galveston, Harris, and Chambers counties. These counties constitute 98% of our study area. Another report from the Office of Homeland Security Division estimated total damages of $9.3 billion by including damages to transportation, public structure, housing, hospital, and infrastructure repairs^35^. NOAA published a report that estimated a higher damage value of $29.5 billion by including all affected states^32^ and Texas Engineering Extension Service published a report that estimated a total loss of $142 billion, which included long-term economic damages^36^. The estimated damage values ($29-$142 billion) in most other reports exceed those in our study because they include damages from several states (Texas, Louisiana, and Arkansas), wind impacts, rain-induced flood damages, and long-term economic impacts. Our study deviates from these reports in that, (1) we focused only on storm surge-induced coastal property damages; (2) assumed that damage only occurs above a minimum (threshold) of flood depth above 0.1 m; (3) assumed that economic damage from storm surge is limited only to buildings and crop lands, and; (4) did not include damages from secondary impacts like business interruption. We also assess the effect of uncertainties in peak flood height estimates on our economic damage values. We find that differences between modelled and observed high water marks result in variations in damage estimates in those hexagons of less than $0.01 million (Fig. S14). We conservatively present all our cumulative results to the nearest $1 million.

The findings from this study highlight the importance of conserving wetland extents, to maintain their flood protection benefits. Several regulations are in place in the Galveston Bay region that protect coastal wetland habitats^38^. In addition, the Galveston Bay region has a long history of wetland conservation efforts by state institutions as well as non-governmental organizations^26^. In 2021, the Galveston Bay Foundation spent ~ $11.5 million obtained from regional and federal grants to acquire over 4,000 acres, or 16 km^2^, of wetland for conservation in Brazoria and Galveston Counties^39^. Based on a unit value of $1.1 million per km^2^, an investment of $12 million in conservation of 16 km^2^ of these wetlands translates to a nearly 150% return on investment on these risk reduction benefits alone, supporting similar findings in other coastal ecosystems and regions^40^. Our estimates of wetland benefits for storm surge damage reduction match well with what previous studies have found though these results vary widely depending on the location and hurricane context. Sun and Carson^41^ estimated the economic value of wetlands during hurricane Irma as $860,000 per km^2^^41^. Other studies in the U.S. North-east have shown the relative benefits of wetlands to vary between 8 and 52%^23^ of total flood damage during individual storm events, and average around 15% of surge damage in terms of annual flood reduction benefits^20^.

The study finds that in a few instances, inter-tidal wetlands also increase storm surge damages, though this effect is small (2.14% of total net benefits) compared to the net positive effect of these wetlands on the rest of the region (Fig. 2a). This finding supports similar findings from previous assessments of the storm surge reduction benefits of tidal marsh and mangrove wetlands^20,42^. This study shows that outer-lying barrier islands in Galveston Bay witnessed an increase in peak flood heights during H. Ike, due to the presence of back-barrier marsh wetlands functioning as semi-permeable barriers that slowed down or blocked the flow of the storm surge further inland. As wetlands become increasingly considered as alternatives within coastal defense portfolios^43^, it becomes essential to understand how these wetlands behave as barriers, and therefore, how they influence flood impacts on areas adjacent to these wetlands.

The findings from this study also highlight the importance of considering changes in wetland extent, real estate value and coastal development when assessing flood risks. Regional land-use management policies, including decisions on land-use planning and conservation, are often updated over 10–30 year timescales^44^. Flood risk management decisions and insurance pricing policies are typically based on static flood maps from the Federal Emergency Management Agency (FEMA) that are updated periodically^45^. However, the process of including these data into insurance policies and zoning laws can take much longer, while the floodplain continues to change in terms of hazards and exposure, potentially rendering these policies outdated on arrival^46^. By quantifying the spatial and temporal variability of storm surge damage risk due to changes in land-use and wetland extent over a decade, our study emphasizes the importance of identifying how changes in these drivers may be influencing present-day flood risk.

The study focuses on investigating storm surge-related flooding and extents and does not consider the effect of waves, making our estimates of both damages as well as wetland benefits somewhat conservative. In shore-front regions, waves can be a significant factor influencing flood extents during a hurricane, potentially increasing damages to people and property^47^. Coastal ecosystems, particularly inter-tidal wetlands have been shown to be highly effective at reducing wave heights during extreme events^48^. Wave-induced flooding and damages, while computationally expensive to model at high resolutions are an important component of flooding and more studies are required to isolate their contribution to flood damages as well as their interactions with coastal wetlands during extreme events. Further research is also required on the benefits of wetlands during high wave events. The assessment of damages uses previously described depth-damage functions that provide empirical information on the extent of structural damage to be expected from different flood depths. These functions are widely used in the place of on-the-ground, observations of structural damage data which can be difficult to obtain after an extreme event [e.g. Ref.^13^].

**Figure 4:**
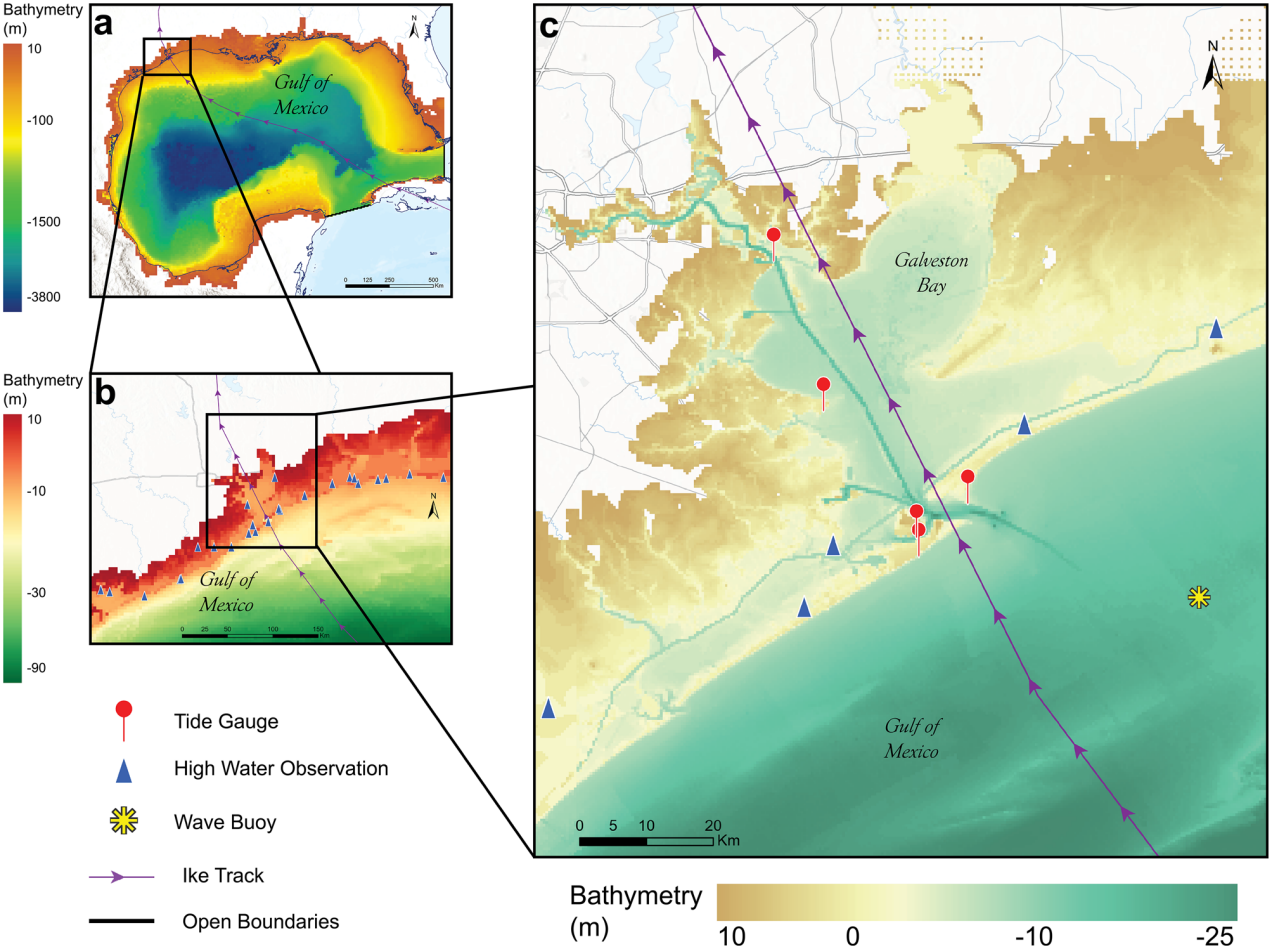
(**a**) Map showing details of the bathymetry (relative to Mean Sea Level (MSL) for Gulf of Mexico (GoM). Black dotted line denotes the open boundaries of the Delft3D, where the tidal forcing is applied. (**b**) Map showing the bathymetry of the northwestern part of GoM where the flood model validation was performed. The USGS high water marks used for model validation are shown in blue triangles. (**c**) Map showing the bathymetry of Galveston Bay and locations of the tidal stations (red circles), high water marks (blue triangle) and wave buoy (yellow star) used for model validation. Arrow line denotes Ike track in all panels.

The simulation of Hurricane Ike’s flooding is performed using a digital elevation model with a resolution of 10 m within the Galveston Bay which captures most of the major shipping and navigation channels (Fig. 4a–c). While most of these channels are captured in the DEM (Fig. 4c), our model has a grid resolution of 86 m, and thus the effect of channels smaller than 86 m in width is averaged within the model. Explicit resolution of smaller channels is computationally expensive for large study regions such as this one but is important for investigation of fine-scale urban flooding in highly channelized urban coastal environments^49^.

Our assessment of wetland loss uses an extreme scenario that assumes that all these wetlands are replaced by open water with no change in the underlying elevation. While extreme, this scenario is the clearest way to explicitly quantify the flood protection benefits of the entirety of these wetlands for storm surge damage reduction. However, such a scenario is not unrealistic for smaller regions, including in the Gulf of Mexico^50,51^. We expect that this estimate is conservative, in that it potentially underestimates wetland benefits, since we do not consider the geomorphological reworking of bathymetry, sediment and currents that could accompany such extensive loss of wetlands^52^, which can further aggravate the increase in storm surge risks.

The population analysis used census data from 2010 which may not reflect the actual population in the region during H. Ike in 2008. The aftermath of Hurricane Ike caused some of the worst-affected regions like Galveston Island to lose population due to people moving away from the island^53^. Evidence from Galveston Island suggests a reduction in population of approximately 11,000 or 5% of the total population within the region flooded by Hurricane Ike’s storm surge^54^. Such migration of people out of a region after an extreme event can cause an underestimation of the total number of people affected during damage assessments. In our study, we used assumptions based on land-cover data from 2008 and 2019, and land-use data from 2019 to fill gaps in land-use data for 2008 for use in the depth-damage functions. These assumptions reduce the accuracy of our damage estimates and highlight the importance of developing, where possible, historic land-use data for better estimates of damage from past hurricanes.

The findings of this study add to a growing literature that shed light on the importance of tidal wetlands for storm surge risk reduction and the simultaneous effect that coastal development has on increasing exposure to these risks. Hurricane Ian in 2022 once again demonstrated the consequences of development choices on risky coastlines, including in front of and behind coastal and inter-tidal mangrove forests^55^. High-resolution, spatially explicit models such as presented in this study can provide critical information to both local planners and restoration practitioners on where to build on coastlines, and where to restore or protect natural habitats as natural coastal defenses.

## Methods

### Study area

Galveston Bay has a length of 50 km, and width of 27 km and is the largest estuary along the Gulf Coast of Texas, covering a surface area of 1554 km^2^. The average depth is 3 m, except where the marine channel passes through the middle of the bay with a depth of more than 12 m (see Fig. 4c). Two rivers (the San Jacinto and Trinity) represent the major sources of freshwater. On the oceanside, two entrances connect the Bay with the Gulf of Mexico. Most human population in the Bay is concentrated in areas on the western side of the Bay. The Bay has coastal wetlands everywhere though most of these are located on the southern side of the Bay, as well some in the eastern and northeastern sides. The total size of the study area in Galveston Bay (land area around Galveston Bay) is 3000 km^2^.

### Hydrodynamic model

DELFT3D-FLOW, a process-based hydrodynamic model, was used for the storm surge flood modeling. The hydrodynamic model only estimates storm surge flows and related flooding and does not account for waves or wave-induced flooding that can occur on top of storm surges. A number of numerical model studies have used different storm surge modeling systems to study the storm surge behavior and inundation in coastal areas in the upper part of the Gulf of Mexico due to Hurricane Ike^50–52^. Veeramony et al.^55^ examined the performance of the Delft3D-FLOW model to simulate storm surge and inundation from Hurricane Ike and found that it predicts the extent of flooding from Ike reasonably well. We only modeled storm surge and did not consider waves in this analysis.

Delft3D-FLOW is a well-developed numerical model commonly used for solving three- or two-dimensional (depth-averaged) unsteady-shallow water equations (continuity [Supplementary Eq. S1] and Reynold-averaged Navier–Stokes equations [Supplementary Eqs.S2 and S3]) on a computational grid with an implicit finite volume approach to simulate coastal hydrodynamic processes^17,53,54^. This study uses Delf3D-Flow in the 2D mode.

An unstructured grid domain with a spatial resolution of 5.5 km (0.05 deg) was assigned to the entire Gulf of Mexico and the resolution increased gradually to a finer grid near the Texas coastline and into the Bay, ultimately reaching a spatial resolution of 86 m within Galveston Bay and the surrounding floodplain. The model domain was built to extend from the outer Gulf of Mexico into the coastal floodplain around Galveston Bay up to a topographical elevation of 10 m. The choice of model resolution of 86 m within Galveston Bay and the floodplain was based on an assessment of the value in terms of flood extents versus the computational costs. Since the 86 m grid cells captured the key landscape features and major navigation channels accurately, we found that increasing this resolution to 30 m did not change the flood extent results significantly but greatly increased the computational costs.

Three bathymetry datasets were integrated into the model (see Table 2). In Galveston Bay, the study used a high spatial resolution bathymetry Digital Elevation Model (DEM) of 10 m. The model included information on tidal constituents implemented as astronomic components in terms of amplitude and phase, obtained from Global Satellite Altimeter data (AVISO) with a spatial resolution of 6.94 km along the two open boundaries at the south and southeast of the Gulf of Mexico domain (black lines in Fig. 4a). To account for changes in water elevations due to seasonal steric expansion in the Gulf of Mexico, an initial water level of 0.125 m was added based on the average seasonal fluctuations in 2008, across 22 Gulf of Mexico tidal stations (Fig. S1). The simulation of storm surge was mainly driven by atmospheric forcing. A spatial and temporal varying wind field dataset was used as external forcing in the model (see Table 2). For wind drag coefficient ${C}_{d}$, the default breakpoints values (${C}_{d}$ of 0.00063 and 0.00723 for ${U}_{10}$ of 0 m/s and 100 m/s, respectively) underestimated the simulated peak water level. We used modified breakpoints (${C}_{d}$ of 0.0028 and 0.0035 for ${U}_{10}$ of 0 m/s and 100 m/s, respectively) to improve model performance in terms of accurately capturing the magnitude and shape of the storm surge peak generated by Ike. The modification of breakpoints set for drag coefficient has been suggested and reported in different studies^10–12^.

**Table 2:** Details of different datasets used for modeling storm surge in Delft3D and the economic analyses.

Dataset	Scale	Type	Purpose	Year	Spatial Resolution (km)	Temporal Resolution
Coastal digital elevation mode DEMs		Bathymetry	Land elevation and Water depth	2007	0.01	–
Coastal relief models CRMs		Bathymetry	Land elevation and Water depth	2001	0.1	–
GEBCO	Global	Bathymetry	Land elevation and Water depth	2021	0.5	
AVISO	Global	Tide	Boundary conditions	2014	6.94	
ERA5 ECMWF	Global	Wind field and barometric pressure	External forcing	2008	55.6	6 h
NLCD	Regional	Land Cover	Friction/damage function	2008/2019	0.03	–
Houston–Galveston area council	Regional	Land Use	Damage function	2019	Property Parcel	
Texas natural resources information system; chambers county official website	State	Land value and improvement value	Damage function	2019	Property Parcel	–
US Census	National	Population		2010/2020*	Census Block	

Dataset were interpolated to match the points of the model grid domain. The references for the datasets are listed in the SI file.

*2020 Census Redistricting Data.

To study the impact of wetlands on storm surge flooding in Galveston Bay, these habitats were represented in the numerical model as Manning coefficients for surface roughness. Coefficient values were derived based on land-cover classifications, obtained from the 2008 National Land Cover Databases NLCD (Supplementary Table S3). The Land Cover classifications were translated into the corresponding Manning’s n value based on Mattocks and Forbes^56^. The focus of this study is on Emergent Herbaceous Wetlands that have Manning’s n value of 0.045. The storm surge model was calibrated using Gulf of Mexico Manning’s n value and wind drag coefficients. Since the areas around the coast of Texas and Louisiana are mostly muddy bottom, a value of 0.012 was used for open water instead of 0.02 which is applicable for sandy areas^55,57^.

### Storm surge validation

To ensure that the Delft3D Flow model accurately captures the tidal signals, the Flow Model was first set up with only tidal components along the open boundaries as external forcing. A total of 34 tidal components were used (see SI file) in the model along with consideration of seasonal variations in water level due to temperature and salinity. The simulation was carried out for a period from Jan 1-Jan 16, 2008 with a 10 min time-step. The statistics of the comparison show a good agreement between the simulated and in situ water level at most of the tide gauge locations, with an R-value ranging from 0.72 to 0.92 and RMSE values between 0.09 m and 0.14 m, although the slope values of the regression line suggest an underestimation of water level (slope 0.85–0.93) (Supplementary Fig. S2 and Table S1). Accurate simulated storm surge modeling is mainly dependent on the accuracy of external forcing especially wind field (wind speed and direction) and the drop in barometric pressure. Thus, the reanalysis of wind field and pressure datasets (from global climate model) were compared with observations at several NOAA tidal stations in Galveston Bay and offshore wave buoys. For wind speeds, the simulations were found to be in good agreement with the observations, where RMS errors smaller than 2.3 m/s with correlation coefficients larger than 0.84 were found. To quantify the wind direction simulation, we determined the complex correlation coefficients. These coefficients range from 0.53–0.83 with angles -7.3 to 3.34 deg. The reanalysis pressure dataset shows robust correlation with observations (Supplementary Figs. S3-S5 and Table S2).

**Figure 5:**
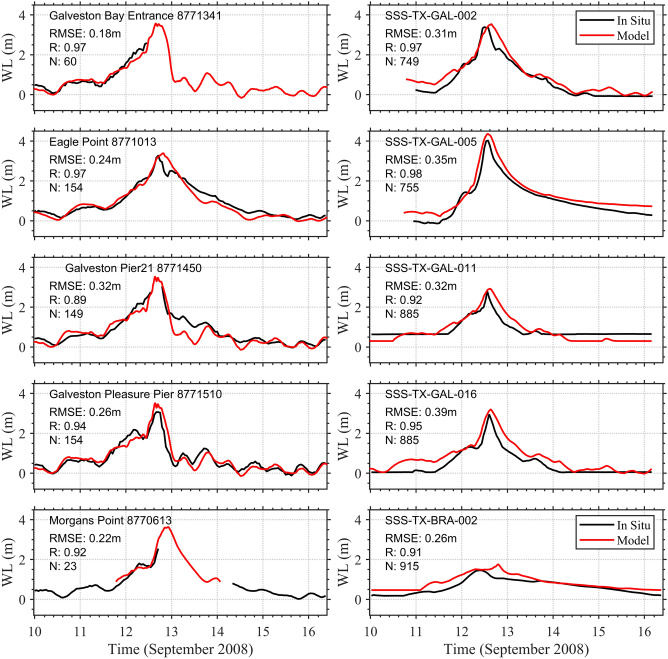
Comparison of total water levels (WL) in Mean Sea Level (MSL) vertical datum between tidal stations (in black) and the storm surge model (in red) at different locations in the study area.

After ensuring the Delft3D model accurately captures the tidal signal, and meteorological observations, the model was setup for storm surge simulation by including the storm wind and pressure field datasets as external forcing. The storm model was run from Aug 20-Sept 29, 2008 to simulate total water level at the shoreline. Figure 5 shows time series comparisons of simulated and measured water level at different tide-gauge locations in Galveston Bay (see left panels in Figs. 4c, 5) as well as several USGS water level gauges (see right panels in Figs. 4b, 5). To quantify the storm surge model performance, statistical analyses are carried out and summarized in Table 3. The storm surge model captures accurately the magnitude and the shape of storm surge peaks at all locations. RMS error values range from 0.18 to 0.39 m with correlation coefficients higher than 0.90 (Table 3).

**Table 3:** Error Statistics for Flood Model. Table shows RMS error (in m), correlation coefficient (r), normalized RMS error (NRMS), regression slope, scatter index (SI), corrected indicator (HH), and bias derived from comparison of simulated water levels and observed water levels at different locations along with the latitude and longitude for each location.

Station	Lon (deg)	Lat (deg)	N	RMSE (m)	r	Slope	NRMS error	SI	HH	Bias
Galveston bay entrance (8,771,341)	− 94.724	29.356	60	0.18	0.97	0.91	0.16	0.16	0.17	− 0.04
Eagle point (8,771,013)	− 94.918	29.480	154	0.24	0.97	0.99	0.20	0.19	0.20	− 0.06
Galveston pier 21 (8,771,450)	− 94.985	29.681	149	0.32	0.90	0.86	0.32	0.30	0.34	− 0.10
Galveston pleasure pier (8,771,510)	− 94.790	29.285	154	0.26	0.94	1.02	0.26	0.26	0.26	0.00
Morgans point (8,770,613)	− 94.793	29.310	23	0.22	0.92	1.06	0.14	0.12	0.14	0.10
SSS-TX-GAL-002	− 95.288	29.084	749	0.31	0.97	1.12	0.27	0.18	0.25	0.23
SSS-TX-GAL-005	− 94.648	29.465	755	0.35	0.98	1.17	0.25	0.14	0.23	0.30
SSS-TX-GAL-011	− 94.390	29.594	885	0.32	0.92	1.06	0.33	0.33	0.32	− 0.01
SSS-TX-GAL-016	− 94.944	29.220	885	0.39	0.95	1.31	0.49	0.30	0.43	0.30
SSS-TX = -BRA-002	− 94.905	29.303	915	0.26	0.91	1.24	0.35	0.21	0.32	0.20

N is the number of records used in comparison.

### Hurricane Ike storm surge flooding scenarios

Once validated, the model was used to calculate storm surge flood extents for three scenarios: (1) 2008 Baseline, with 2008 mean-sea level (MSL), and land-cover; (2) 2008 No Wetlands 2008, with 2008 MSL, and land-cover but no wetlands (here wetlands are replaced by open water); and (3) 2019 “Present-day Hurricane Ike”, with 2019 MSL, 2019 land-cover. Scenario 3 was conducted to investigate the effect of land cover/land-use changes including wetland loss, and sea-level rise on storm surge flood damages.

In scenario (3), the storm surge model for Hurricane Ike was modified in terms of wetland cover (Manning’s n value of friction coefficient) from 2019 land cover data and run for the period from August 31, 2019 to September 18, 2019. Here, sea level rise was considered by adding the average value of SLR from number of tidal stations (0.0582 m over 11 years) to the initial water level in addition to the increase in water level due to average seasonal fluctuations in September 2019 of 0.125 m. These values were calculated based on the sea level trend from tidal gauges in Gulf of Mexico within 11 years (from 2008 to 2019).

For each scenario spatially explicit maps of peak flood extents were produced by considering total water levels from the Delft-3D model that are above MSL and subtracting the elevation of the underlying topography. A minimum peak flood threshold of 0.1 m was considered for mapping flooded areas^58^.

### Economic damage analyses

Spatially explicit model results of flood extents and heights for each scenario were integrated with publicly available data on property type and value to assess the economic value of storm surge damage for each scenario. We did this in five steps using the modelled flood heights and publicly available socio-economic data: (1) determine peak flood heights; (2) determine land-use and property type; (3) determine total property value; (4) determine population; (5) map property value on to depth-damage functions that relate flood heights to the expected extent of structural damage^59^. Due to the variation in spatial resolutions across the flooding, property value and property type datasets, all economic analyses were performed on hexagons of uniform size with a side of 86 m and area of 11,320 m^2^, with these dimensions chosen such that each hexagon contains between two and four flood height data points. All dollar amounts were converted to US$ 2019 values.

First, the flood height for each hexagon was estimated as the average of the flood height point values within the hexagon boundary. Then, land-use and property type were determined in the flooded regions for years 2019 and 2008. Land-use data for 2019 were obtained from datasets of the Houston–Galveston Area Council for all areas that intersect with flooded extents from the model. From these datasets, we classified 2019 land-use within the floodplain into six categories: Residential, Commercial, Industrial, Agricultural, Infrastructure and Undeveloped (see Section SI 4, Supplementary Fig. S8). These land-use categories were used to map flood depths to damage extents within each hexagon. Each hexagon was assigned a land-cover and land-use according to the category that has the largest area within the hexagon.

Since there is no publicly available information about land-use in the region for 2008, land-cover data for 2019 and 2008 were used to examine temporal changes in land-cover, and then to predict a land-use category for 2008 for each hexagon. First, land-cover data from USGS for both years were reclassified into three categories: (1) Developed, (2) Farmland, (3) Undeveloped. If land-cover remained the same between 2008 and 2019, we assumed that land-use in 2008 was the same as in 2019. For all areas where land-cover had changed between 2008 and 2019, we assigned land-use categories based on the 2008 land-cover data (see Section SI 4). We found that 18,615 hexagons, i.e. ~7%, experienced a change in land-cover between 2008 and 2019, out of a total of 264,995 hexagons. All changes in land-cover between 2008 and 2019 represented an increase in development (i.e. change from undeveloped to developed, or farmland to developed), with the exception of 14 hexagons, which we ignored.

Once land-use and property type were determined, the next step was to obtain total property value. Property appraisal tax data were collected from each county, that include a given property’s parcel land value and improvement value. The land value here refers to the value of the vacant plot. The improvement value refers to the value of the building and other additional structures, calculated as the difference between the total real estate market price and the land value. For aggregation with the hexagonal units of analysis, land and improvement values in each parcel were first converted to values per square meter. Then, the ‘Union’ function in ArcGIS Pro was used to obtain the proportion (area) of each parcel within a given hexagon. Based on this, the land and improvement values for each hexagon were estimated as the linear area-weighted sum of all land-use classifications within each hexagon (Supplementary Fig. S7). The final property value for each hexagon was then determined based on land-use. For all land-use types with structures, the total property value was set equal to the improvement value. Structures on undeveloped land, such as parks, were assumed to be infrastructure (i.e. public use buildings, etc.), and structures on agricultural land were assumed to be residential (i.e. homes). Out of the 165,492 hexagons, 40,539 don’t have property value. The total property value of all hexagons within the study domain is $28.74 billion for 2008 and $40.8 billion for 2019 (both in 2019 US dollars). For agricultural land-use hexagons, where flood damage predominantly affects the land itself, the total property value was set equal to the sum of land value and improvement value. Agricultural land value was estimated per acre based on the median of all available agricultural land values from the 2019 tax data in a given county (see Section SI 4), multiplied by the total acreage of farmland in the given hexagon (Supplementary Fig. S9).

The calculation of 2008 economic values also accounted for changes in median real estate values between 2008 and 2019. Due to lack of publicly available property appraisal tax data in 2008, changes in development between 2008 and 2019 were used to determine development ratios for estimating 2008 property values (Section SI 4; Fig. S11). Changes in development were examined for all hexagons between 2008 and 2019. For all hexagons, if there was a change in development for a particular hexagon, the 2019 improvement value was adjusted by the ratio of development change to obtain the 2008 improvement value. For all agricultural hexagons, median agricultural land value per acre from 2019 was applied to the total acreage farmland in each hexagon. Then, ratios of regional housing price and rural land values between 2008 and 2019 were applied to all 2008 improvement and land values to capture real estate value changes between 2008 and 2019.

Population for each hexagon was determined using data from the 2010 Census and 2020 Census Redistricting datasets at the census block geographic level (US census). Like property value data, population data were calculated per square meter and then rejoined with each hexagon to obtain population per hexagon. We assumed that the land cover and population don’t change for the short periods of time between 2008–2010 and 2019–2020.

Storm surge damages to properties were then estimated by integrating the peak flood heights with the total property value for all hexagons. For each hexagon we applied depth-damage functions that estimate the proportion of flood damage based on the average flood height, dominant property type and total property value associated with that hexagon. We used the depth-damage functions described in Huizinga et al.^59^ with a regression fitting analysis to convert the binned depth-damage data in Huizinga et al.^59^ to a continuous function applicable at any flood depth (Supplementary Fig. S12). Additionally, we counted the number of people flooded within each hexagon as the population within hexagons where the average flood depth is greater than 0.1 m. Thus, spatially explicit values for economic damage and people flooded were obtained based on the damage from peak flood depths for all storm surge scenarios. All dollar amounts were converted to 2019 dollars.

Finally, from the damage estimates, we calculated wetland benefits as the difference between the 2008 Baseline and 2008 No Wetlands damages. The combined decadal effect of urban development, i.e. change in land-use type and value, and wetland change were calculated as the difference between the 2019 “Present Day. Hurricane Ike” scenario and the 2008 Baseline scenario. For the decadal analysis, we estimated two damage values, one accounting for the increase in median real estate values between 2008 and 2019, and one without accounting for this change. Median real estate values for both years were obtained from public data on median house and rural land prices^60^.

## Supplementary Information

Supplementary Information.

## Data Availability

The MATLAB scripts for preparing the input data for the model simulation and analyzing the results will be available online on GitHub (https://github.com/Zaidrahman85/Storm_Surge_Analysis_Code) and Zenodo (https://zenodo.org/record/7631246#.ZBM5o3bMK3A).
