# Supplementary material for: The impacts of tidal wetland loss and coastal development on storm surge damages to people and property: a Hurricane Ike case-study
Source: Sci Rep. 2023 Mar 21;13:4620. doi: 10.1038/s41598-023-31409-x (PMC10030854; doi:10.1038/s41598-023-31409-x)
Supplement: Supplementary file 1 — Supplementary Information. [file 41598_2023_31409_MOESM1_ESM.docx]

Supplementary Information file

The impacts of tidal wetland loss and coastal development on storm surge damages to people and property: a Hurricane Ike case-study

# Zaid Al-Attabi, Yicheng Xu, Georgette Tso and Siddharth Narayan

# SI 1. Model description

The 2D version of Delft3D was used for storm surge and water level simulations [1, 2] in this study. We used the 2D model with an unstructured grid domain to simulate coastal surges induced by meteorological forcing during Hurricane Ike in 2008. The model uses equations of momentum and continuity to estimate the volume of water in each grid cell.

$$\frac{\partial\zeta}{\partial t}+\frac{\partial\left( d+\zeta\right)u}{\partial x}+\frac{\partial\left( d+\zeta\right)v}{\partial y}=0 (S1)$$

$$\frac{\partial u}{\partial t}+u\frac{\partial u}{\partial x}+v\frac{\partial u}{\partial y}+fv=\frac{\rho_{a}C_{d}\left( U_{10}-u \right)}{\rho\left( d+\zeta\right)}{\left[ {(U}_{10}-u \right)^{2}+\left( V_{10}- v \right)^{2}]}^{\frac{1}{2}}-\frac{\rho gn^{2}}{d^{\frac{1}{3}}}u\sqrt{u^{2}+v^{2}}+v_{H}\left| \frac{\partial^{2}u}{\partial x^{2}}+\frac{\partial^{2}u}{\partial y^{2}} \right|-g\frac{\partial\zeta}{\partial x} (S2)$$

$$\frac{\partial v}{\partial t}+u\frac{\partial v}{\partial x}+v\frac{\partial v}{\partial y}-fu=\frac{\rho_{a}C_{d}\left( V_{10}-v \right)}{\rho\left( d+\zeta\right)}{\left[ {(U}_{10}-u \right)^{2}+\left( V_{10}- v \right)^{2}]}^{\frac{1}{2}}-\frac{\rho gn^{2}}{d^{\frac{1}{3}}}u\sqrt{u^{2}+v^{2}}+v_{H}\left| \frac{\partial^{2}v}{\partial x^{2}}+\frac{\partial^{2}v}{\partial y^{2}} \right|-g\frac{\partial\zeta}{\partial y} (S3)$$

Where $u$ and $v$ are depth averaged velocities, $\zeta$ is water surface elevation (above still water), $d$ is water depth (above still water), $f$ is the Coriolis parameter, $g$ is the gravitational acceleration, $\rho_{a}$ and $\rho$ are air and water densities, $v_{H}$ is the horizontal viscosity coefficient, $C_{d}$ is the wind drag coefficient, $U_{10}$ and $V_{10}$ are the wind velocities at 10 m above the water level, and $n$ is the Manning coefficient. The model computes the water level and flow from appropriate initial conditions, tidal forcing for boundary conditions, wind stress and atmospheric pressure on the water surface, and other forcing from density and pressure gradients.

An unstructured grid domain was created for the entire Gulf of Mexico using Delft3D grid editor RGF-GRID [3]. The vertical datum of bathymetry data was transformed to common datum (MSL) using NOAA vertical datum transformation tool (VDatum v4.4) [4].

The bathymetry data [5, 6, 7] was interpolated to match the number of unstructured grid cells using Delft3D Flexible Mesh Suite HM (2021.03) Delta shell. The averaging interpolation method was used since the resolution of bathymetry data is higher than the resolution of unstructured grid (see Fig. 4 in the main text).

A total of 34 tidal components (K1, M2, M4, N2, O1, P1, Q1, S1, S2, K2, 2N2, E2, J1, L2, La2, M3, M6, M8, Mf, MKS2, Mm, MN4, MS4, MSf, MSqm, Mtm, Mu2, N4, Nu2, R2, S4, Sa, Ssa, and T2) were used [8]. The water elevation in Gulf of Mexico is subject to seasonal fluctuations, mainly due to the variations in water temperature and also salinity. Therefore, analysis of averaged seasonal cycle of mean sea level in Gulf of Mexico was carried out by using water elevations of 22 tidal gauges across the Gulf in 2008. The averaging steric expansion shows variation depending on the season (see Fig. S1). The average increase in water level for Gulf of Mexico in September 2008 is calculated to be 0.125 m. This initial water level value was added to the tidal forcing condition.

Meteorological data from ERA5 ECMWF reanalysis global data was used for wind and atmospheric pressure inputs [9]. This dataset provides spatial and time varying wind vector and atmospheric pressure data with spatial and temporal resolutions of (0.5 degree and 6 hours respectively). Gridded meteorological data for the entire Gulf of Mexico in 2008 was used for model simulations. The momentum exchange from the atmosphere (wind) to the ocean surface water was determined by the wind drag coefficient $C_{d}$ . Wind drag coefficient used in this study is based on wind speed data. $C_{d}$ was calculated based on two breakpoints velocity (linearly varying of wind drag coefficient with wind speed at elevation of 10 m $U_{10}$) as suggested by Smith and Banke (1975) and described in Deltares (2021a) [10, 11]. The default breakpoints values ($C_{d}$ of 0.00063 and 0.00723 for $U_{10}$ of 0 m/s and 100 m/s, respectively) underestimated the simulated peak water level. We used modified breakpoints ($C_{d}$ of 0.0028 and 0.0035 for $U_{10}$ of 0 m/s and 100 m/s, respectively) to improve model performance in terms of accurately capturing the magnitude and shape of the storm surge peak generated by Ike. The modification of breakpoints set for drag coefficient has been suggested and reported in different studies [12, 13, 14].


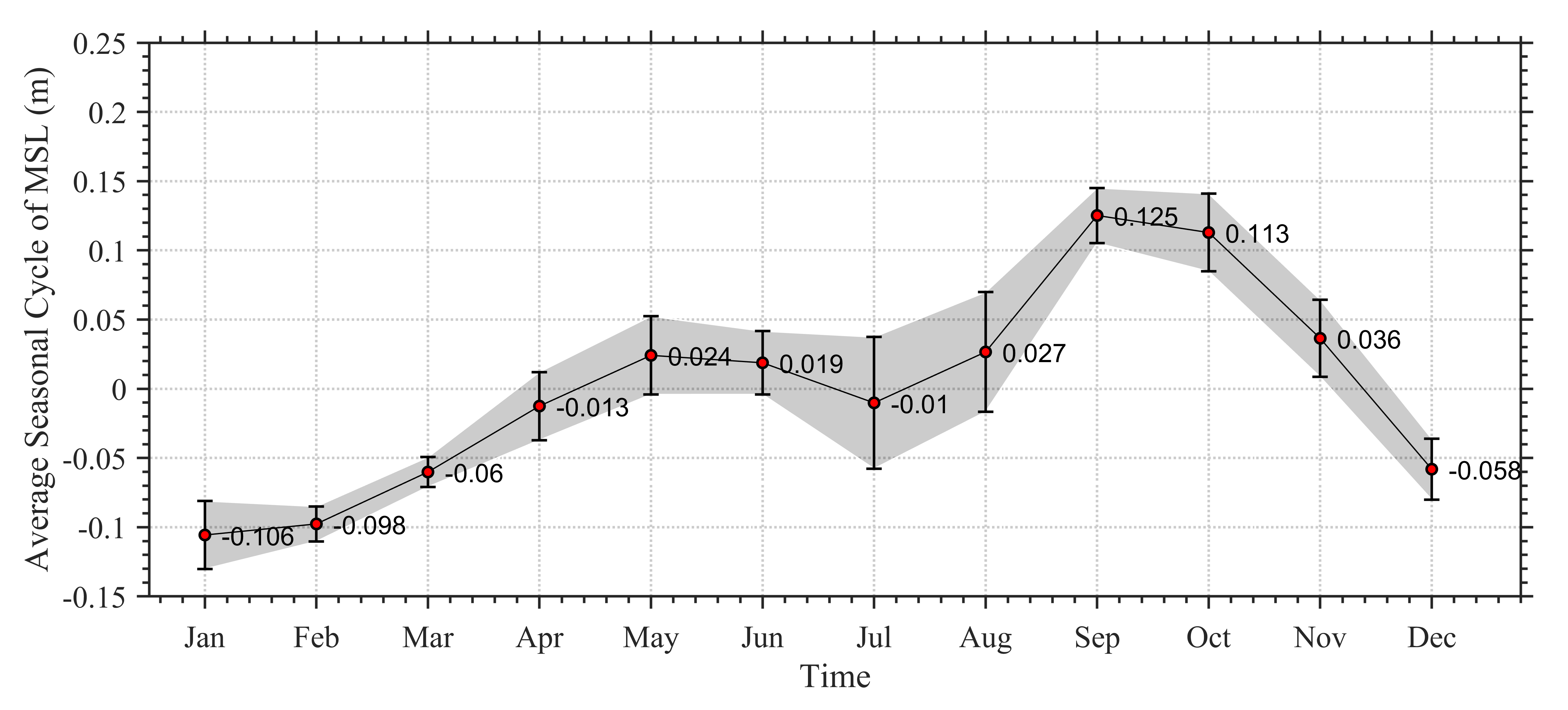


**Figure S1.** Monthly averaging steric expansion across 22 tidal stations in Gulf of Mexico. Initial water level of 0.125 m (in September 2008) was used for the model calibration.

Land cover data from the National Land Cover Database (NLCD) [15, 16] was used and converted to a roughness value (Manning’s n value) based on Mattocks and Forbes (2008) [17]. The spatial varying Manning’s coefficient was interpolated using an averaging method provided by Delft3D-FM to generate a roughness map that matched the grid domain. We found that the lowest Manning’s value (0.012) and wind drag coefficient were the only parameters that influenced model accuracy during the calibration of the storm surge simulation. This aligns with findings from Salehi’s study (2008) of hurricane storm surge modeling in the lower Delaware bay [12].

# SI 2. Simulated Water level (Tide only)

The validation of simulated water levels was carried out by comparing the simulated water level values to those from the observation tide gauges levels at different locations in Galveston Bay (see Fig. 4 in the main text). Several statistical parameters were determined to quantify the agreement between simulated and observed water levels, including root mean square error (RMSE), linear correlation coefficient (r), slope of regression line, and bias. Because the RMS error is always dependent on the magnitude of the water level conditions, we also calculated the normalized RMS error (NRMS error) and Scatter Index (SI). Some studies have reported that these parameters may not sufficiently describe model performance accuracy, especially when negative bias values are reported, thus the corrected indicator used by Hanna and Heinold (1985), the HH indicator, was also considered [18]. To test whether the Delft3D Flow model accurately captured the tidal signals, the model was setup with only tidal components along the open boundaries as external forcing. This simulation run was carried out for the period Jan 1st to Jan 16th, 2008 with a 10min time-step. The time series comparisons between the simulated and measured water levels for a period between Jan 4th – 16th 2008 are shown in Fig. S2. The statistics of the comparison are listed in Table S1. There is good agreement between the simulated and in situ water levels at all four tide gauge locations, where the R value ranges from 0.72 to 0.92 and RMSE values are between 0.09 m and 0.14 m, although the slope values of the regression line suggest underestimation of water level (Slope 0.85-0.93). This underestimation is understandable as wind field data is not included in this run (see Table S1).

| **Station** | **Lon (deg)** | **Lat (deg)** | **N** | **RMSE (m)** | **r** | **Slope** | **NRMS**  **error** | **SI** | **HH** | **Bias** |
| --- | --- | --- | --- | --- | --- | --- | --- | --- | --- | --- |
| Galveston Bay Entrance  (8771341) | -94.724 | 29.356 | 276 | 0.09 | 0.90 | 0.88 | 0.44 | 0.44 | 0.47 | 0.001 |
| Eagle Point  (8771013) | -94.918 | 29.480 | 276 | 0.13 | 0.72 | 0.85 | 0.83 | 0.83 | 0.90 | -0.009 |
| Galveston Pier 21  (8771450) | -94.985 | 29.681 | 276 | 0.14 | 0.80 | 0.93 | 0.77 | 0.75 | 0.79 | 0.030 |
| Galveston Pleasure Pier  (8771510) | -94.790 | 29.285 | 276 | 0.09 | 0.92 | 0.88 | 0.4 | 0.40 | 0.42 | 0.004 |

**Table S1.** List of statistical parameters from comparison of simulated water level and in situ. RMS error (in m), correlation coefficient (r), normalized RMS error (NRMS), regression slope, scatter index (SI), corrected indicator (HH), bias (BI) and N is the number of records used in the comparison.


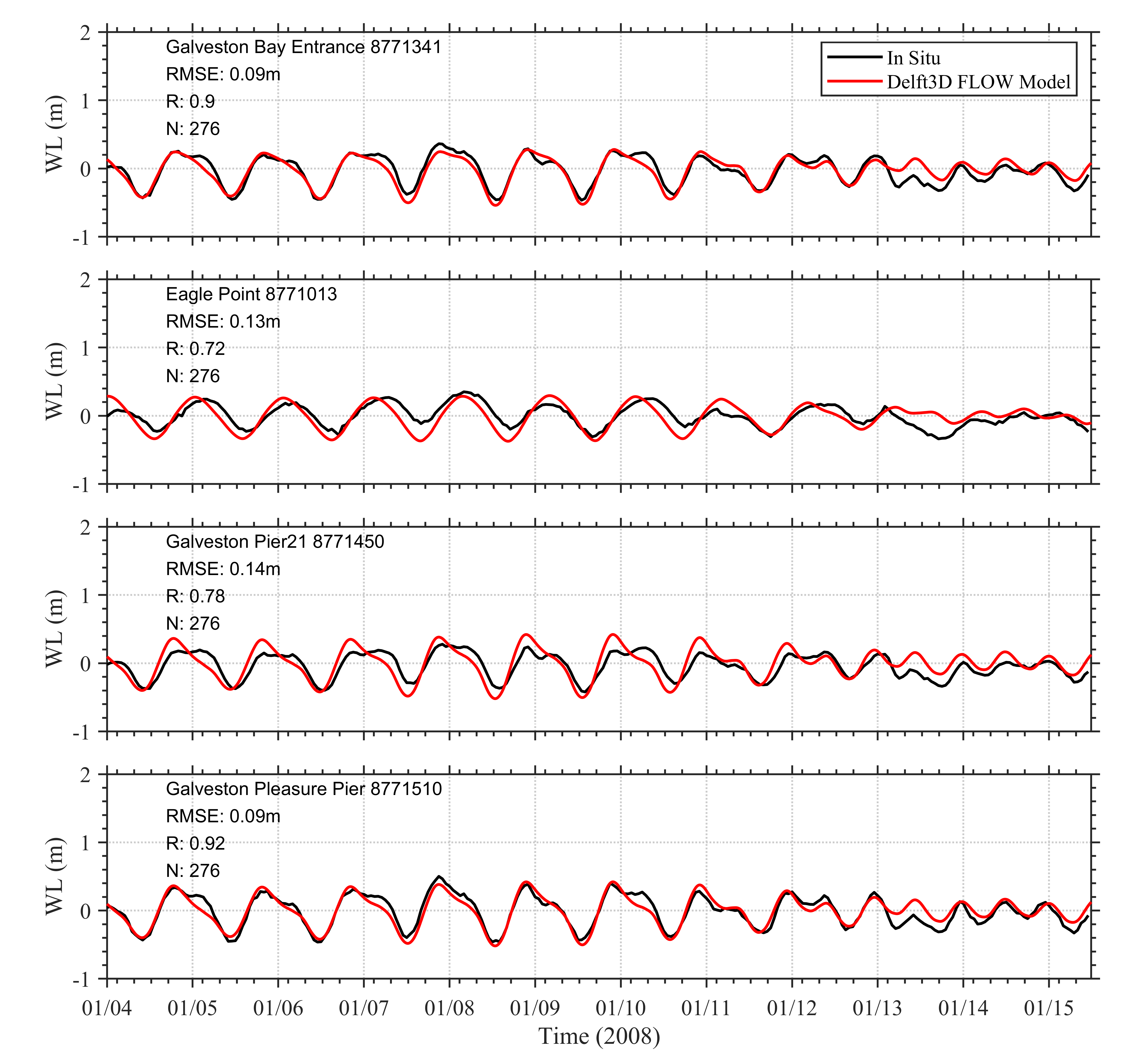


**Figure S2.** Time series comparison of measured (black) and simulated (red) water levels (WL in Mean Sea Level vertical datum) at different locations within the study area. Measured water levels are obtained from tidal gauges. Simulations of water level is achieved using tidal forcing only.

# SI 3. Evaluation of the modeled meteorological data

| **Station** | **Lon (deg)** | **Lat (deg)** | **N** | **Wind Speed** | | **Wind Direction** | | | **Bar. Pressure** | |
| --- | --- | --- | --- | --- | --- | --- | --- | --- | --- | --- |
|  |  |  |  | **RMSE (m/s)** | **r** | **RMSE (m/s)** | **Complex r** | | **RMSE (Pa)** | **r** |
|  |  |  |  |  |  |  | **\|r\|** | Angle (deg) |  |  |
| Galveston Bay Entrance  (8771341) | -94.724 | 29.356 | 59 | 1.5 | 0.98 | 33 | 0.02 | 146 | 39 | 1 |
| Eagle Point  (8771013) | -94.918 | 29.480 | 104 | 3.3 | 0.92 | 73 | 0.90 | -18 | 188 | 0.99 |
| Morgans Point (8770613) | -94.793 | 29.310 | 66 | 1.5 | 0.94 | 18 | 0.85 | 1.2 | 93 | 1 |
| Wave buoy (NDBC 42035) | -94.413 | 29.232 | 154 | 3.0 | 0.89 | 59 | 0.81 | 2.3 | 221 | 0.99 |

**Table S2.** Statistical comparisons of simulated and observed wind direction, wind speed, and barometric pressure at all tidal stations in Galveston Bay and an offshore wave buoy. Values of Root Mean Square (RMS) error and complex correlation coefficient magnitude |r| and angle are listed; N is the number of data points used in the comparisons.


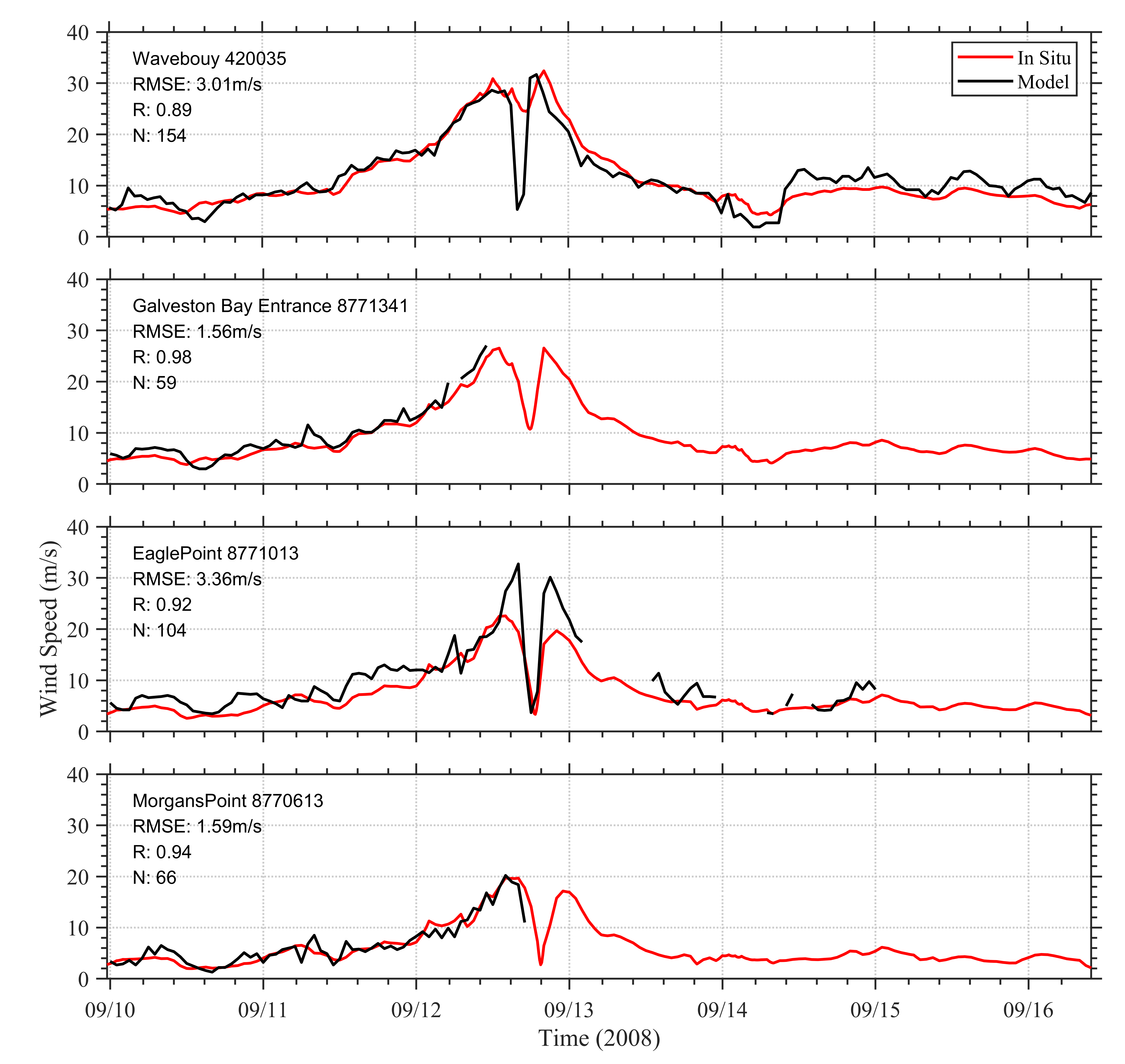


**Figure S3.** Time series comparisons of simulated (black) and observed (red) wind speeds at different locations in Galveston Bay during Hurricane Ike in 2008.


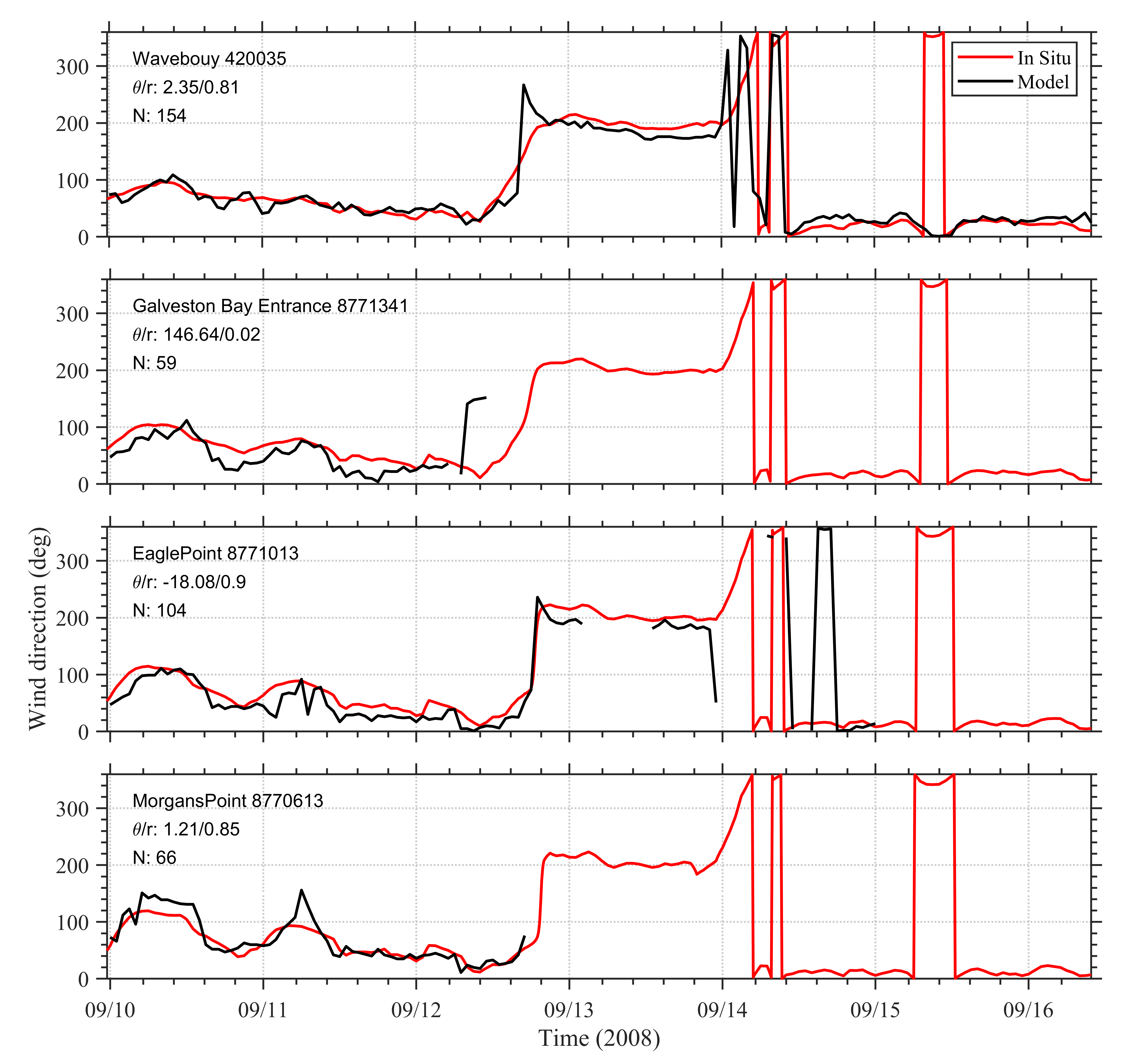


**Figure S4.** Time series comparisons of simulated (black) and observed (red) wind direction at different locations in Galveston Bay during Hurricane Ike in 2008.


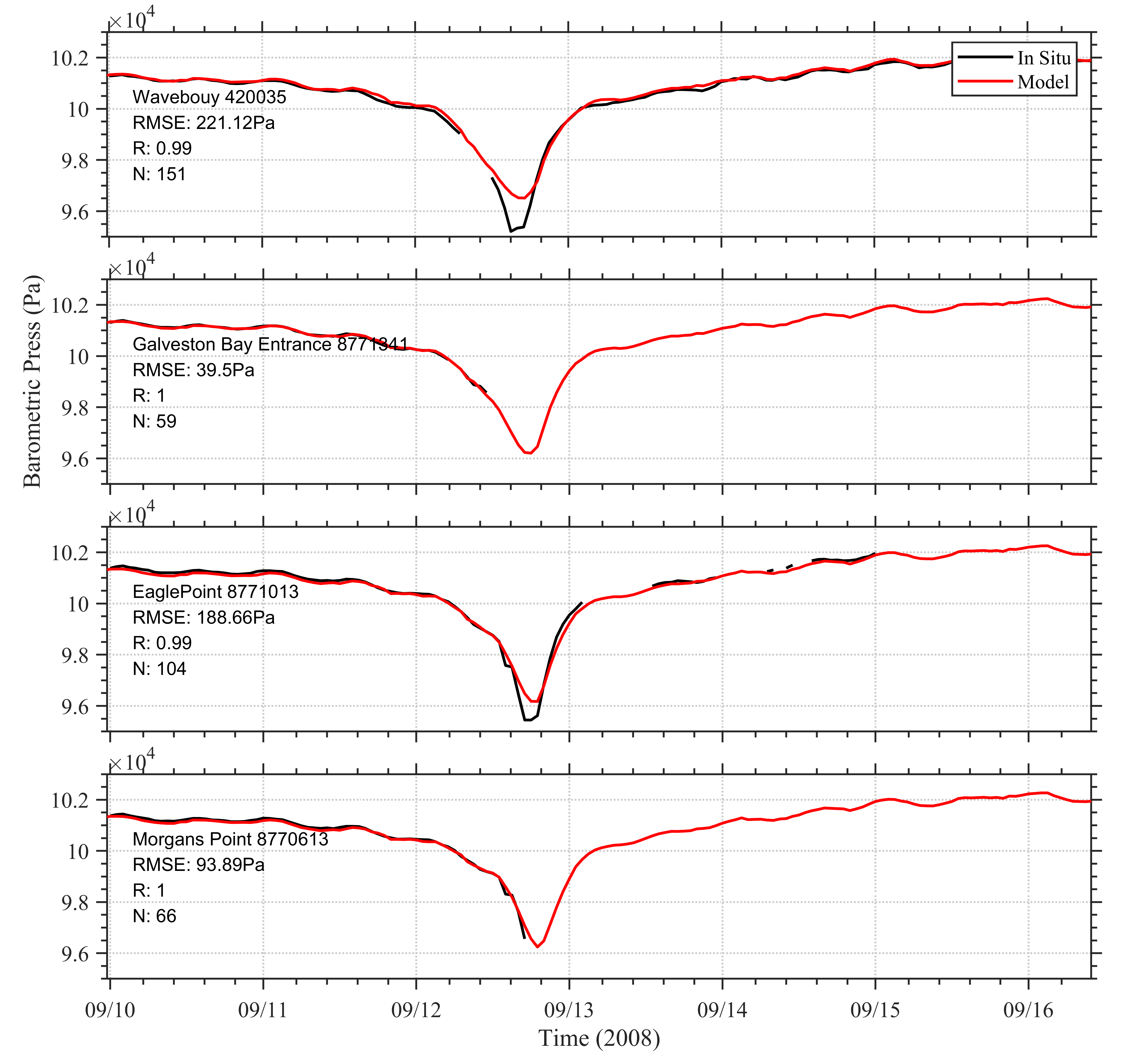


**Figure S5.** Time series comparisons of simulated (black) and observed (red) barometric pressure at different locations in Galveston Bay during Hurricane Ike in 2008.


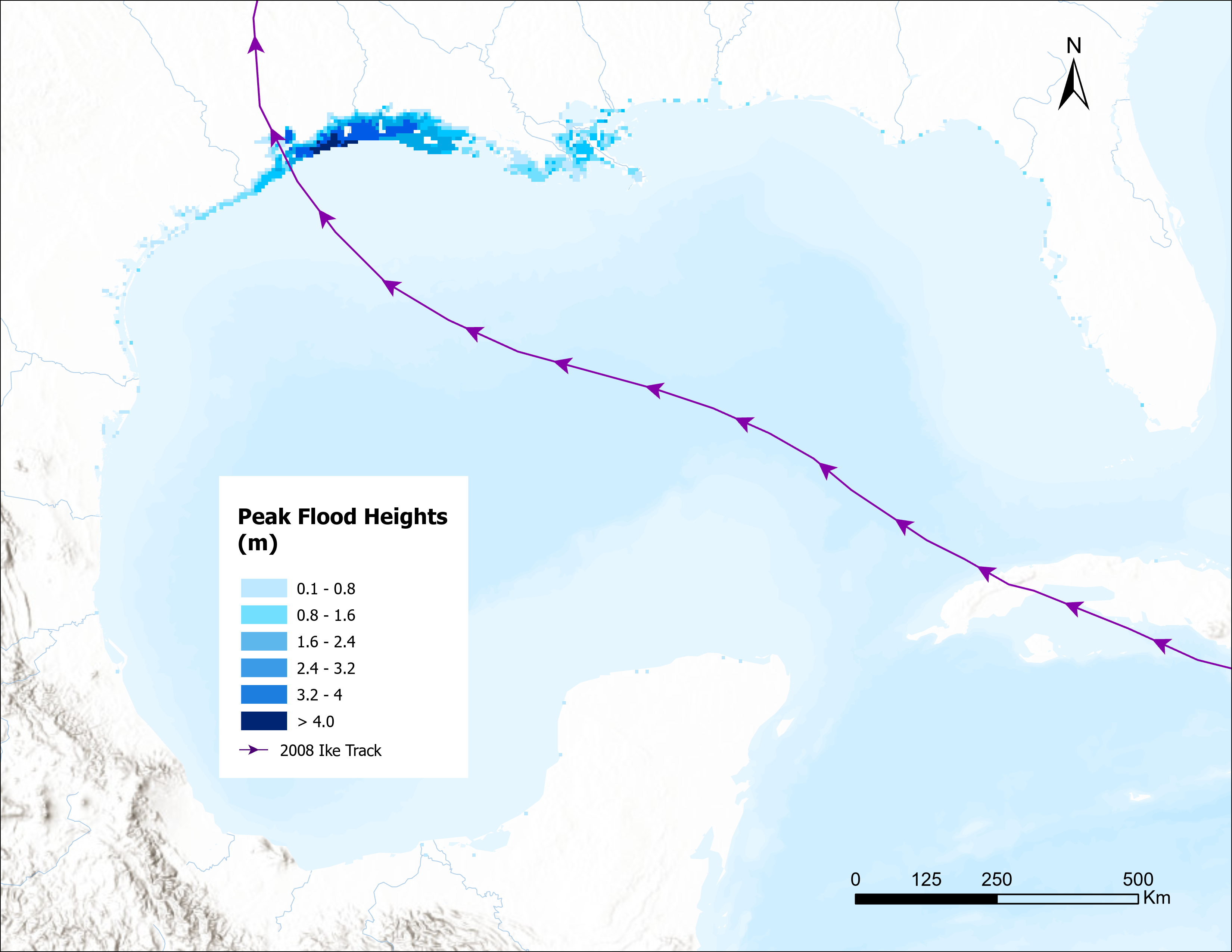


**Figure S6.** Map showing the extent of peak flood heights due to storm surge during Hurricane Ike in 2008 in the study area. Purple line shows the track of Hurricane Ike.

| **NLCD Class Number** | **NLCD Class Name** | **Manning’s n friction coefficient** |
| --- | --- | --- |
| 21 | Developed, Open Space | 0.020 |
| 22 | Developed, Low Intensity | 0.050 |
| 23 | Developed, Medium Intensity | 0.100 |
| 24 | Developed, High Intensity | 0.150 |
| 31 | Barren Land | 0.090 |
| 41 | Deciduous Forest | 0.100 |
| 42 | Evergreen Forest | 0.110 |
| 43 | Mixed Forest | 0.100 |
| 52 | Shrub/Scrub | 0.050 |
| 71 | Herbaceous | 0.034 |
| 81 | Hay/Pasture | 0.033 |
| 82 | Cultivated Crops | 0.037 |
| 95 | Emergent Herbaceous Wetlands | 0.045 |
| 90 | Woody Wetlands | 0.100 |
| 11 | Open Water | 0.020 |

**Table S3.** Manning’s n friction coefficient for NCLD land cover classification [Based on study by Mattocks and Forbes (2008) [17].

# SI 4. Economic analysis

##

**
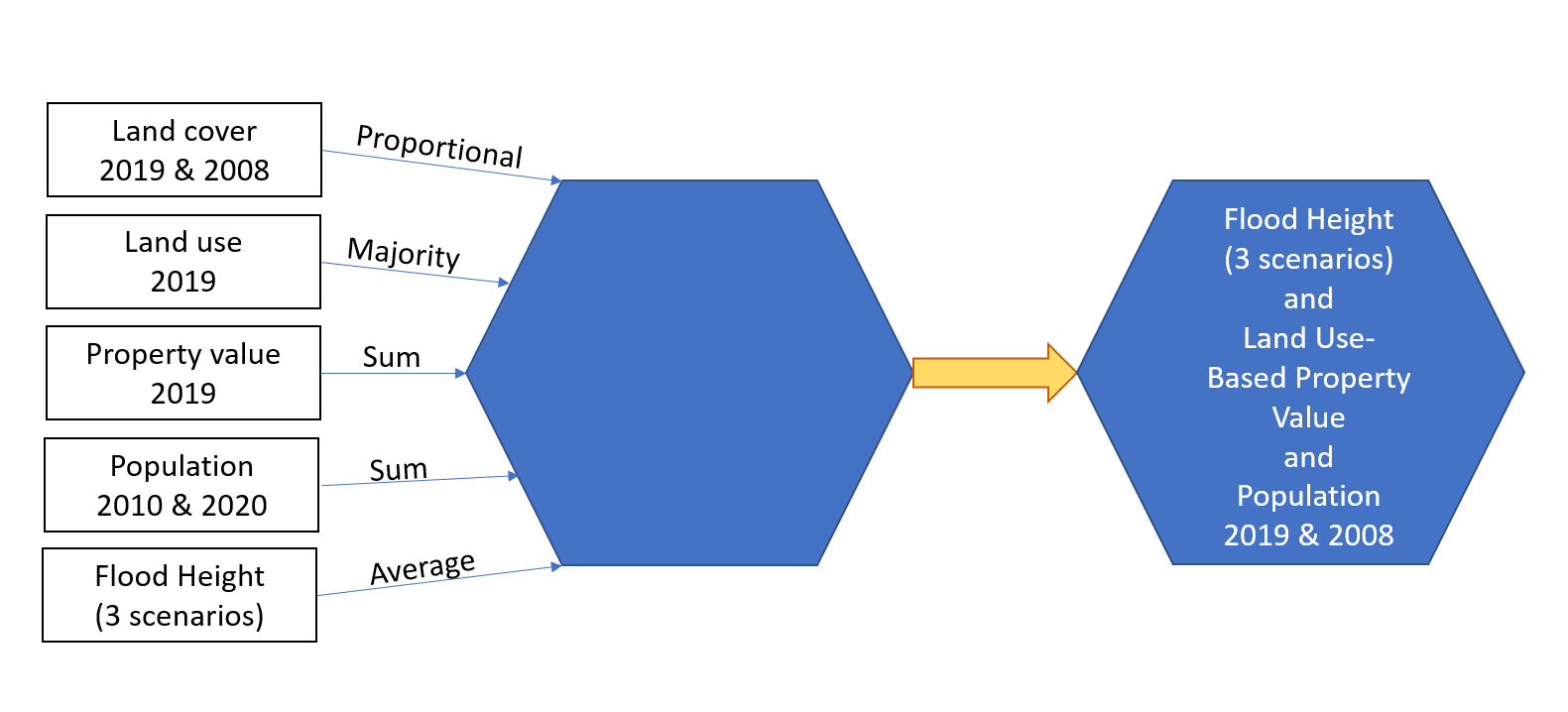
**

**Figure S7.** Schematic of methods used to obtain flood height, land use-based property value, and population for each hexagon in 2008 and 2019.

Land-use data from 2019 was reclassified into six total land-use categories (Residential, Commercial, Industrial, Agricultural, Infrastructure, and Undeveloped) according to the reclassification scheme detailed in Supplementary Fig S7.


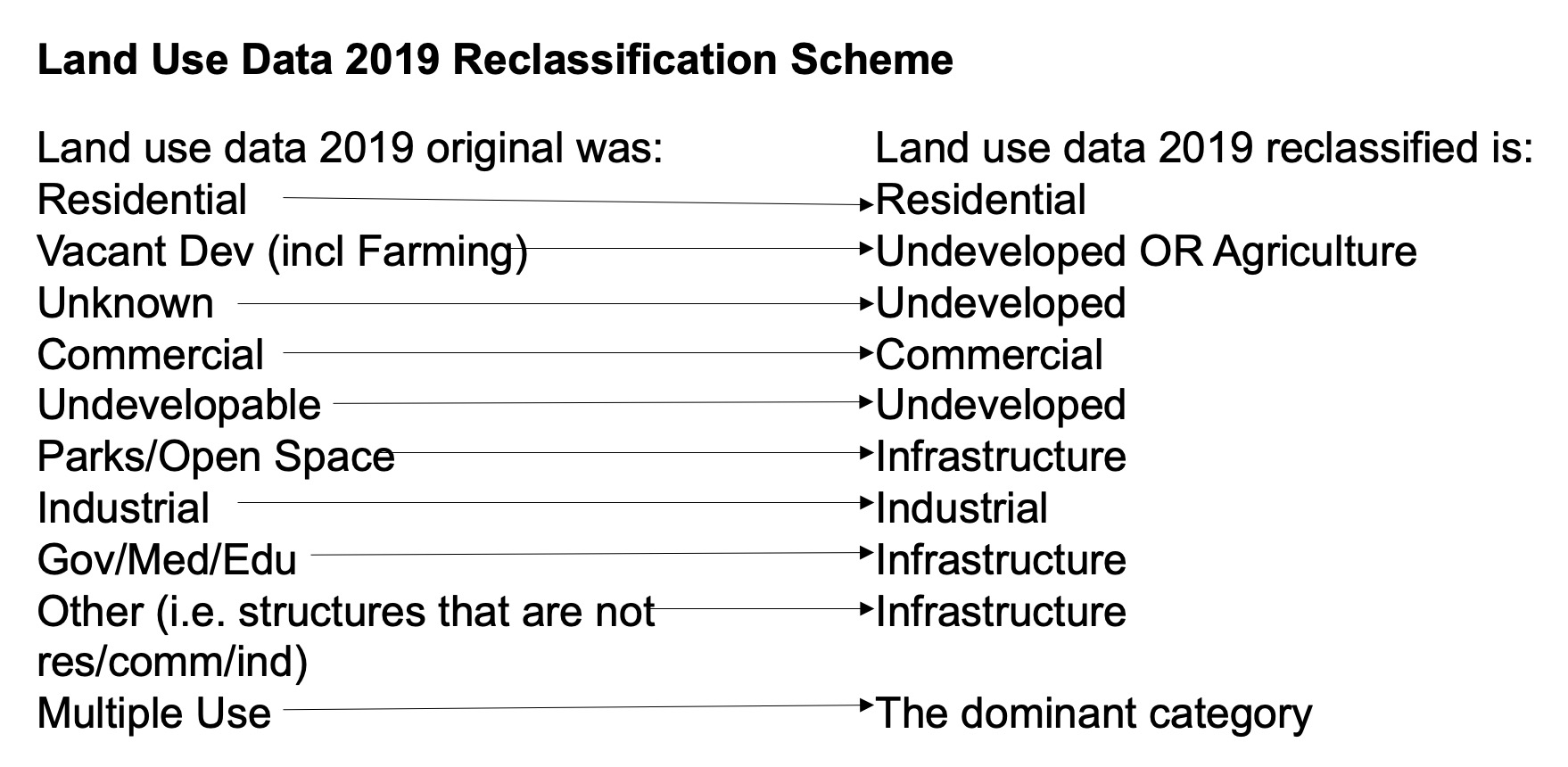


**Figure S8**. Reclassification scheme for 2019 land-use data.

The updated land-use categories were used to determine how property value data was applied to parcels within each hexagon (Supplementary Fig. S8). Property values were separately considered as improvement values and land values. For all hexagons, improvement values were summed. Improvements on agricultural land are assumed to be residential structures, while improvements on undeveloped land are assumed to be infrastructure (eg. park infrastructure). Only hexagons that included agricultural land used land values as part of total property value, since agricultural land is assumed to sustain damage from flooding exceeding just damage to built structures (eg. damage to crops). Median farmland value per county was applied to total area of agricultural land within each hexagon and summed with improvement value to estimate total property value for agricultural land. For all other land-use categories, the total property value was the sum of the improvement values for their respective land-use categories.


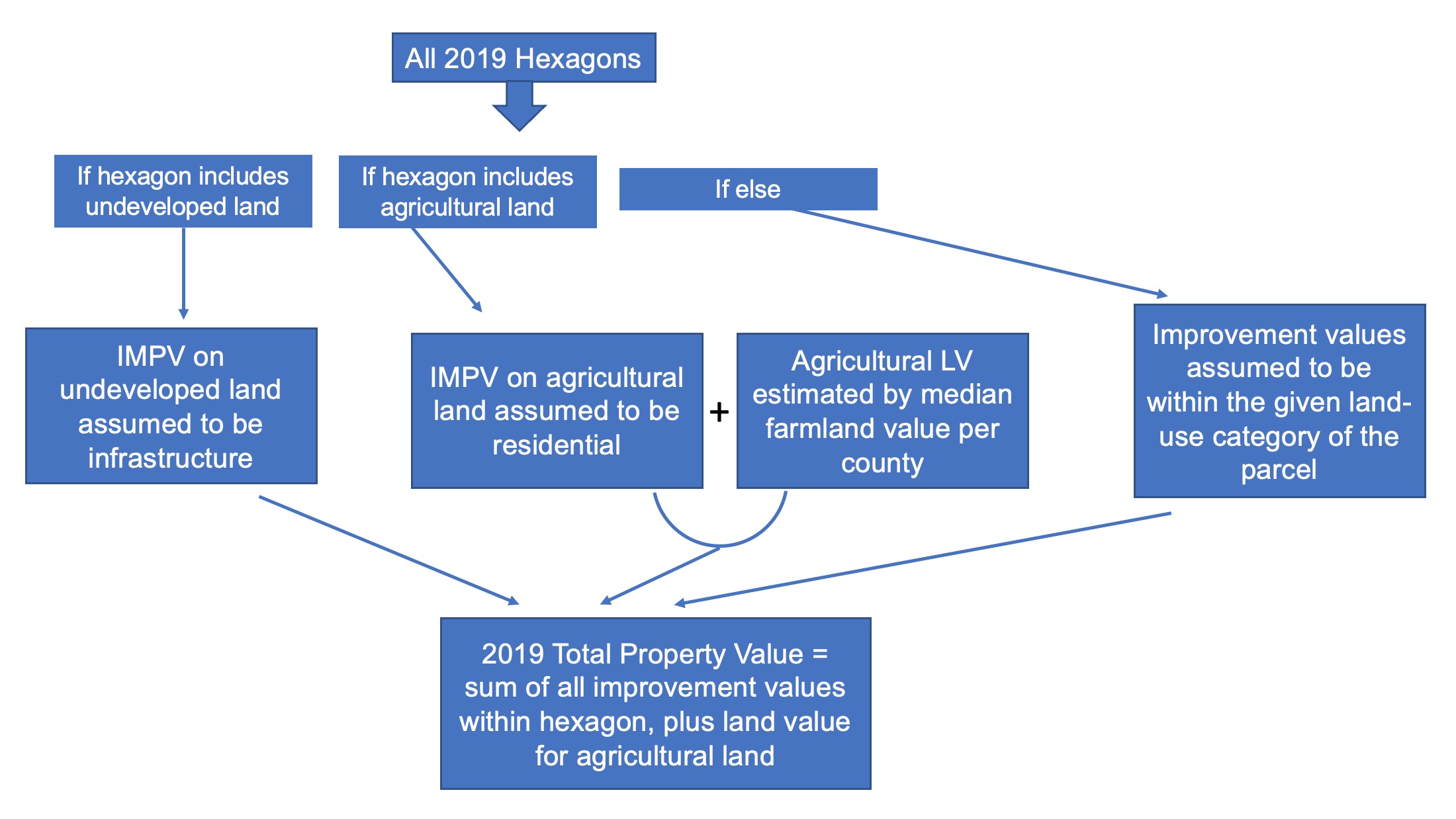


**Figure S9.** Decision tree for determination of Total Property Value (TPV) for each 2019 hexagon.

Since there is no publicly available information about land-use in the region for 2008, land-cover data for 2019 and 2008 was used to examine temporal changes in land-cover, and then to predict a land-use category for 2008 hexagons. For all areas where land-cover has changed between 2008 and 2019, we assign land-use categories based on the 2008 land-cover data according to temporal changes in land-cover between 2008 and 2019, as described in Supplementary Fig. S9.

If the land cover in 2019 and in 2008 is dominated by development, the hexagon was assumed to have the 2019 land use in 2008 (this was the case for 11 hexagons out of 264,995 hexagons). If the land cover in 2019 is not dominated by development, these hexagons were thrown out due to lack of data available to make assumptions about 2008 land use (this was the case for 3 hexagons out of 264,995 hexagons).

The determination of 2008 land-use was also used in the determination of 2008 total property value per hexagon. The development ratio was calculated by the land cover change rate (% land cover change in 2019 -% land cover change in 2018). The real estate market change ratio for all hexagons was calculated by dividing the average median housing price in 2008 by the average median housing price in 2019 (ratio = 0.76) [19]. The real estate market change ratio for agricultural land within hexagons was calculated by dividing the median rural land price in 2008 by the median rural land price in 2019 (ratio = 0.89) [20].


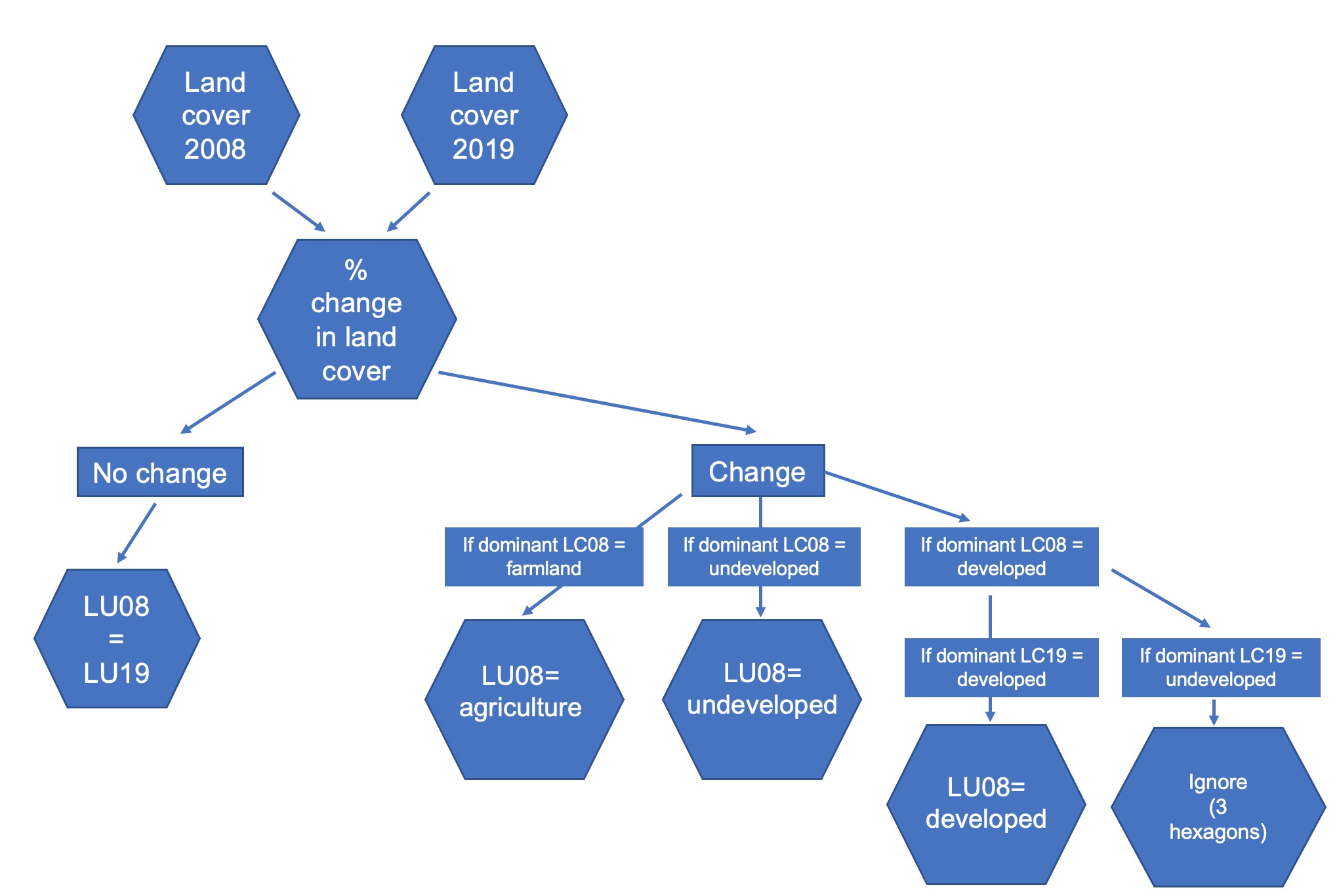


**Figure S10.** Decision tree for determination of land-cover for each 2008 hexagon, based on 2008 and 2019 land-cover data, and 2019 land-use data.

**
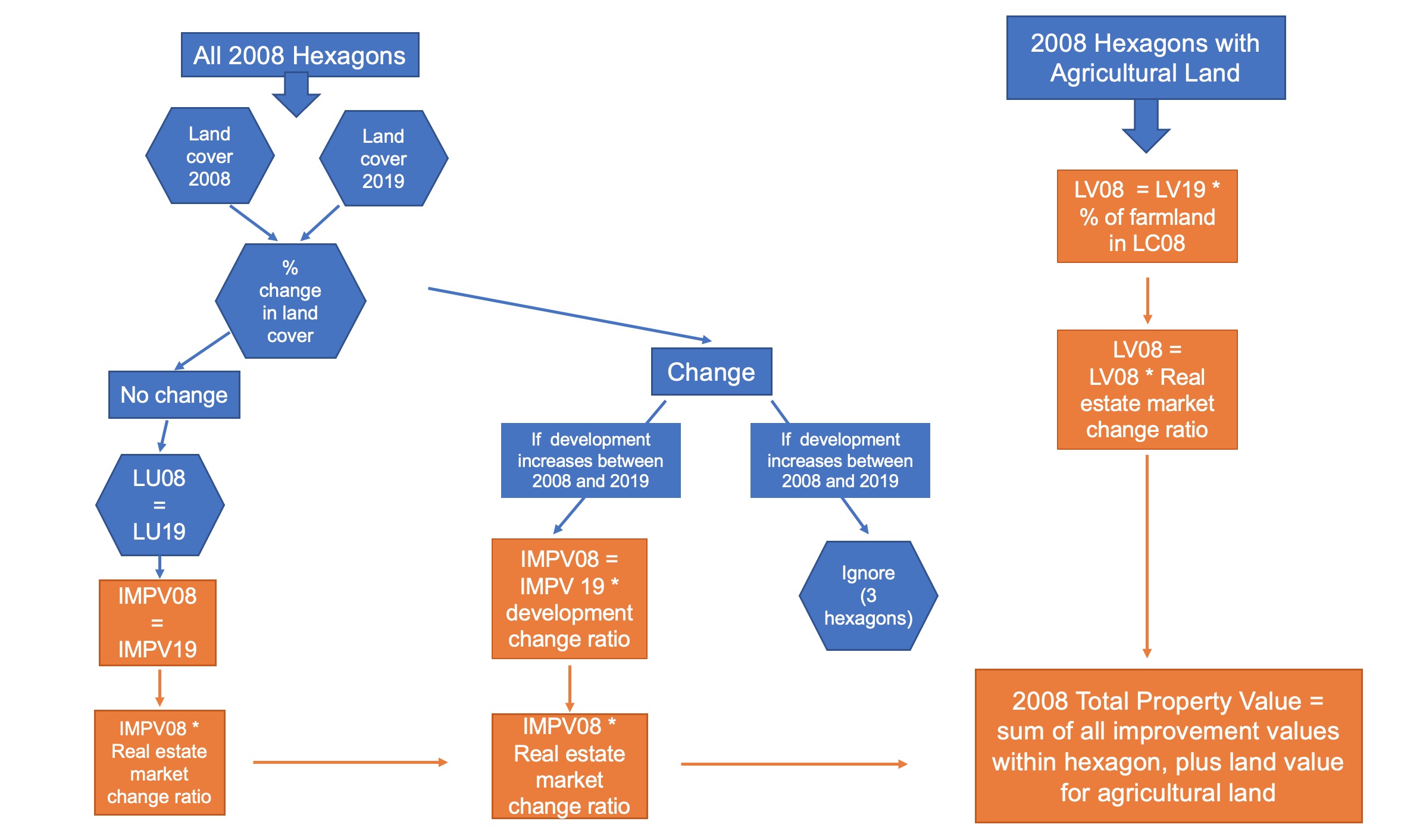
**

**Figure S11.** Decision tree for determination of Total Property Value for each 2008 hexagon.

# SI 5. Flood Damage analysis and Model Validation


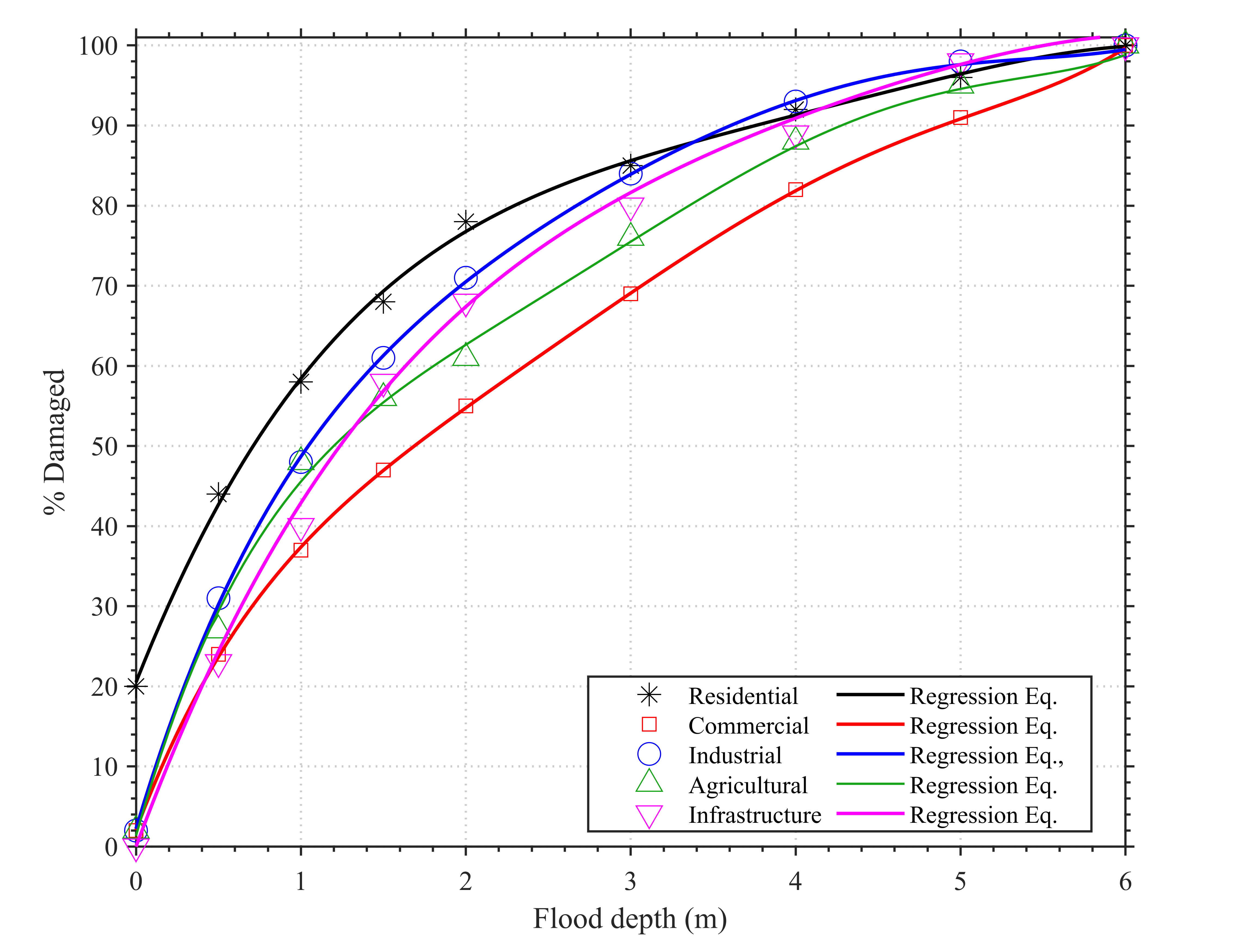


**Figure S12.** Flood damage functions (percentage of damage as function of Flood depth) for five land use classes. The colored shaped symbols denote the table data reported in Huizinga et al (2017) [21]. The colored curves show the regression functions for all five classes based on the table data.


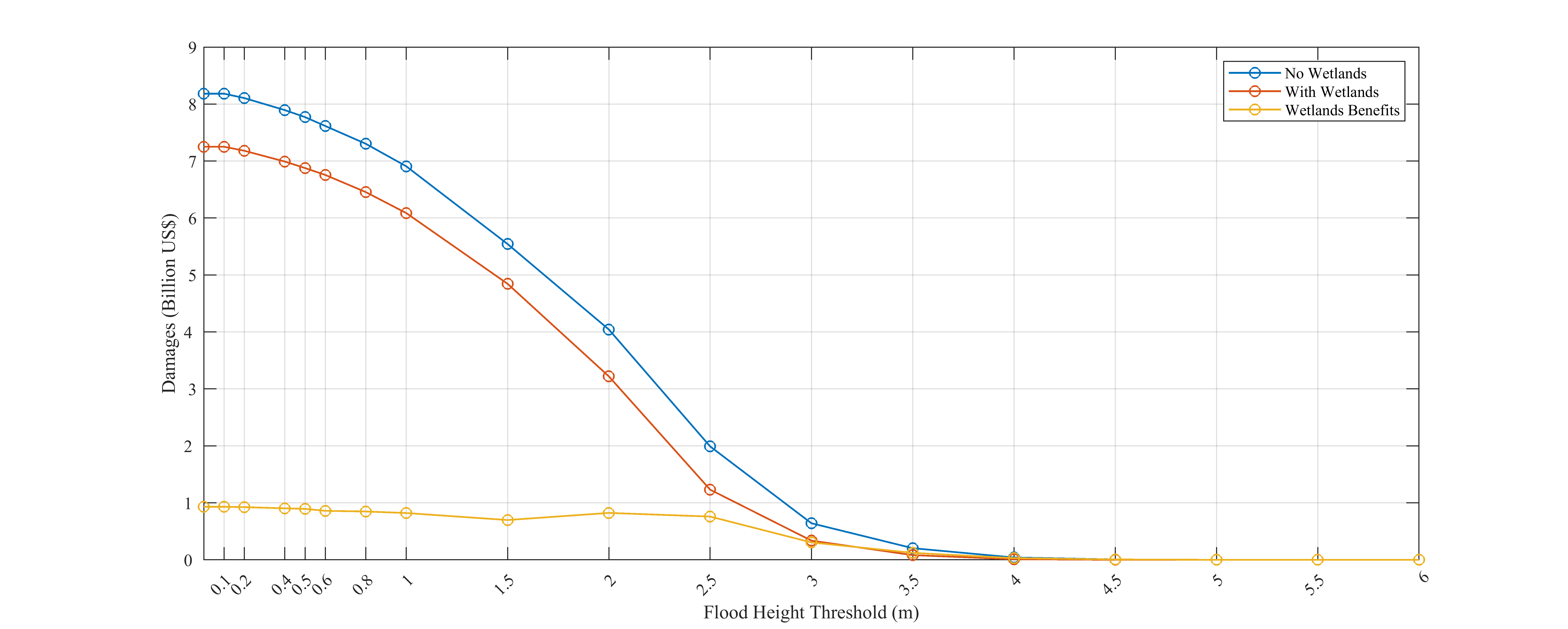


**Figure S13.** Total damage value as function of flood height threshold for scenarios: 2008 Baseline and 2008 No Wetlands.

| **Flood heights (m)** | **Population** | | | **Area (km^2^)** | | |
| --- | --- | --- | --- | --- | --- | --- |
|  | **Without 2008** | **With 2008** | **With 2019** | **Without 2008** | **With 2008** | **With2019** |
| <0.1 | 243661 | 262132 | 310503 | 1212 | 1324 | 1326 |
| 0.1-0.8 | 38737 | 36226 | 39067 | 209 | 208 | 209 |
| 0.8-1.6 | 47555 | 45346 | 50688 | 312 | 289 | 290 |
| 1.6-2.4 | 42399 | 45113 | 51678 | 367 | 378 | 378 |
| 2.4-3.2 | 29463 | 15257 | 19018 | 285 | 287 | 285 |
| 3.2-4 | 3128 | 1062 | 1319 | 253 | 328 | 327 |
| >4 | 787 | 584 | 518 | 361 | 185 | 185 |

**Table S4.** Classification of peak flood heights for all three scenarios and corresponding values of total area and population for different flood height ranges.


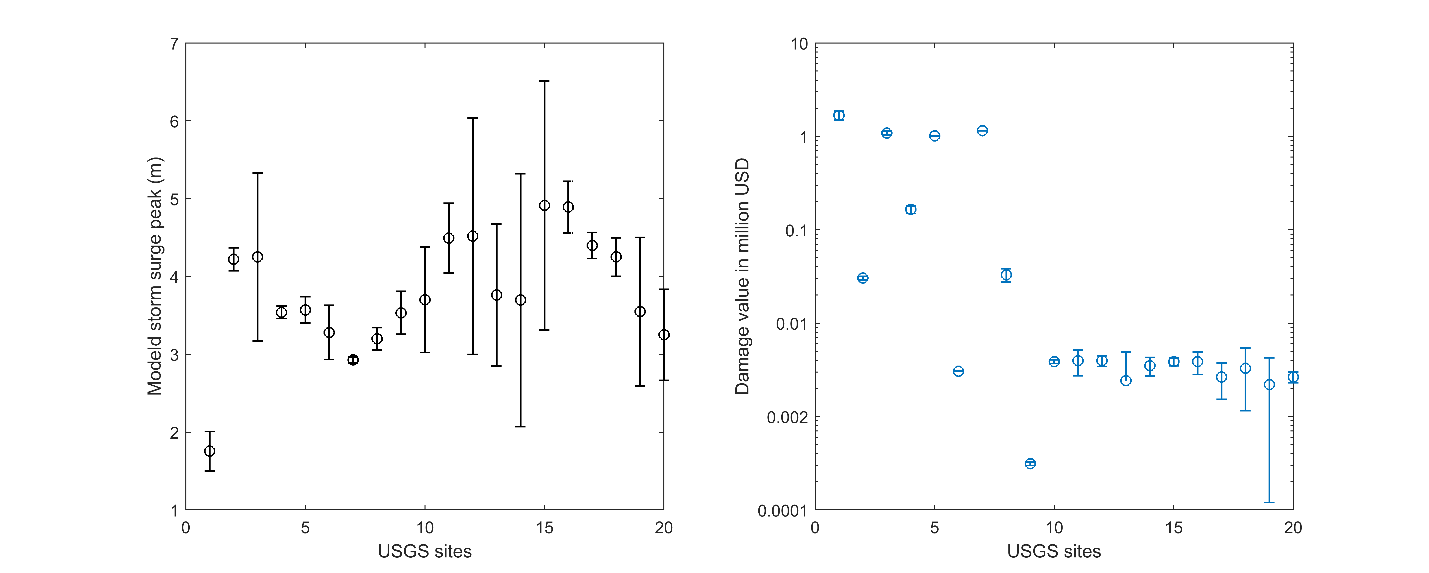


**Fig. S14**. Effect of uncertainty in flood heights on flood damage estimates. Left-hand panel shows the modelled peak flood height and 95% confidence interval range based on observed flood heights at 20 USGS high water marks within the model domain. Right-hand panel shows the modelled damage value and range arising from the storm surge values in the left-hand panel at these locations.


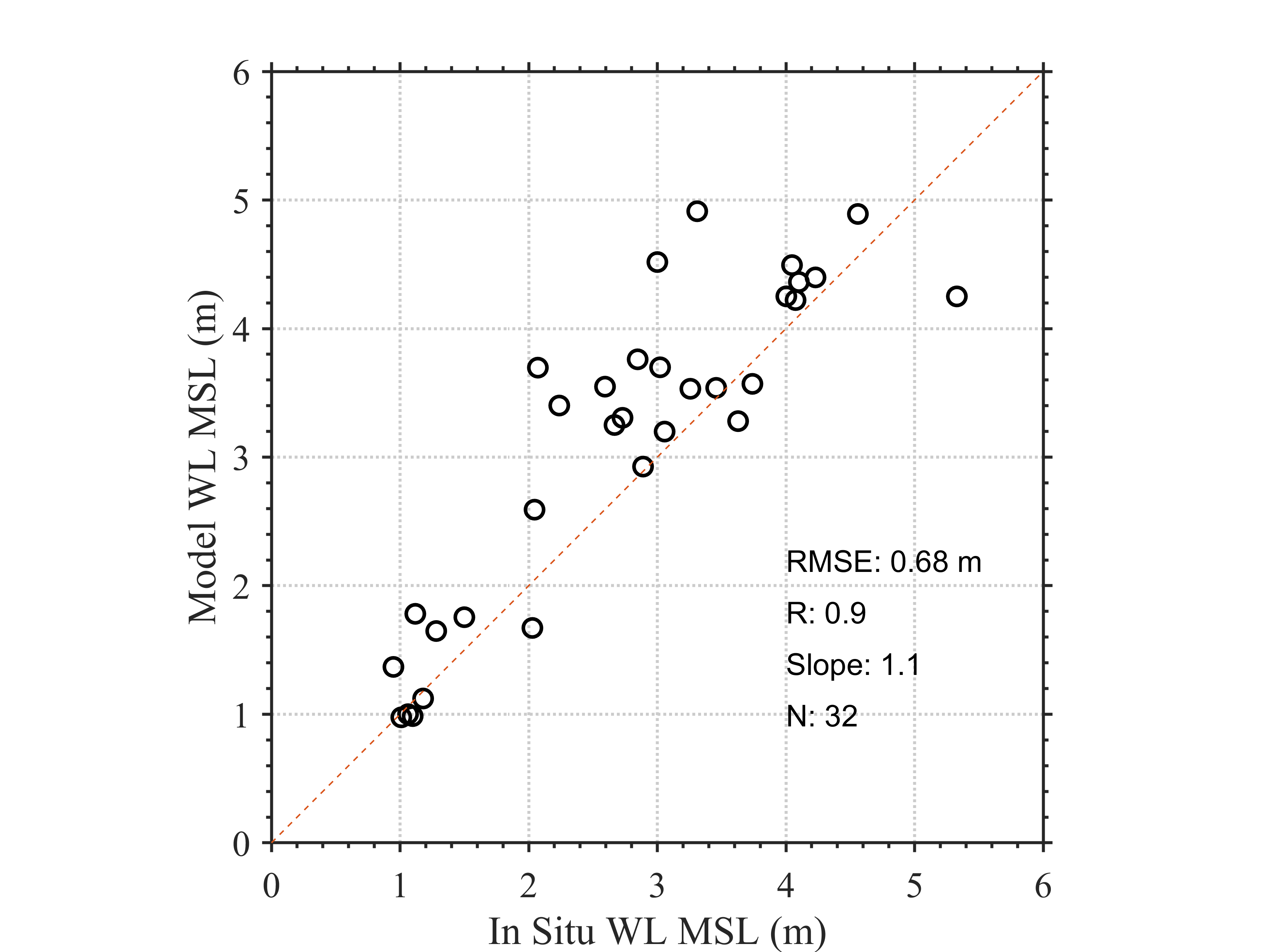


**Fig. S15**. Comparison of observed USGS High Water Marks (x-axis) and Modelled Peak Flood Heights (y-axis) within the H. Ike floodplain.

# References

1. Horstman, E., Dohmen-Janssen, M. and Hulscher, S.J.M.H. Modeling tidal dynamics in a mangrove creek catchment in Delft3D. In *Coastal Dynamics 2013 Conference*, 833-844 (2013).
2. Lesser, G.R., Roelvink, J.V., van Kester, J.T.M. & Stelling, G.S. Development and validation of a three-dimensional morphological model. *Coastal Engineering* **51**, 883-915 (2004).
3. Deltares, RGFGRID generation and manipulation of structured and unstructured grids, suitable for Delft3D-FLOW, Delft3D-WAVE or D-Flow Flexible Mesh. User Manual, Version 5.00., 145 (2021b).
4. Parker, B., Milbert, D., Hess, K. & Gill, S. National VDatum–The implementation of a national vertical datum transformation database. In *Proceedings from the US Hydro 2003 Conference*, 24-27 (2003).
5. NOAA National Geophysical Data Center. Galveston, Texas Coastal Digital Elevation Model. NOAA National Centers for Environmental Information. At https://www.ncei.noaa.gov/access/metadata/landing-page/bin/iso?id=gov.noaa.ngdc.mgg.dem:403 (2007).
6. NOAA National Geophysical Data Center. U.S. Coastal Relief Model Vol.4 - Central Gulf of Mexico. NOAA National Centers for Environmental Information. At <https://doi.org/10.7289/V54Q7RW0> (2001).
7. GEBCO Compilation Group. Grid available at doi:10.5285/c6612cbe-50b3-0cff-e053-6c86abc09f8f (2021).
8. Global Satellite Altimeter data/Global tidal models (AVISO). Altimetry data used in this study were developed, validated, and distributed by the CTOH/LEGOS, France <https://www.aviso.altimetry.fr/en/data/products/auxiliary-products/global-tide-fes.html>
9. Hersbach, H., *et al*. The ERA5 global reanalysis. *Q. J. R. Meteorol. Soc.***146**, 1999-2049 (2020).
10. Smith, S.D. & Banke, E.G. Variation of the sea surface drag coefficient with wind speed. *Q. J. R. Meteorol. Soc.* **101**, 665-673 (1975).
11. Deltares, Delft3D-Flow, simulation of multi-dimensional hydrodynamic flows and transport phenomena, including sediments: user manual, Version 3.15, 718. (2021a)
12. Salehi, M. Storm surge and wave impact of low-probability hurricanes on the lower Delaware bay—Calibration and application. *J. Mar. Sci. Eng.* **6**, 54 (2018).
13. Bennett, V.C. & Mulligan, R.P. Evaluation of surface wind fields for prediction of directional ocean wave spectra during hurricane sandy. *Coastal Engineering* **125**, 1-15 (2017).
14. Fan, Y. & Rogers, W.E. Drag coefficient comparisons between observed and model simulated directional wave spectra under hurricane conditions. *Ocean Modelling* **102**,1-13 (2016).
15. Dewitz, J., and U.S. Geological Survey, National Land Cover Database (NLCD) 2019 Products (ver. 2.0, June 2021). At <https://doi.org/10.5066/P9KZCM54> (2021).
16. Homer, C.G., *et al*. Conterminous United States land cover change patterns 2001–2016 from the 2016 National Land Cover Database. *ISPRS Journal of Photogrammetry and Remote Sensing* **162**, 184–199. At <https://doi.org/10.1016/j.isprsjprs.2020.02.019> (2020).
17. Mattocks, C. & Forbes, C. A real-time, event-triggered storm surge forecasting system for the state of North Carolina. *Ocean Modelling* **25**, 95-119 (2008).
18. Hanna, S.R. & Heinold, D.W. Development and application of a simple method for evaluating air quality models (No. 4409). American Petroleum Institute (1985).
19. Texas Real Estate Research Center, Texas A&M University. Housing Activity for Houston-The Woodlands-Sugar Land. At [https://www.recenter.tamu.edu/data/housing-activity/#!/activity/MSA/Houston-The_Woodlands-Sugar_Land](https://nam02.safelinks.protection.outlook.com/?url=https%3A%2F%2Fwww.recenter.tamu.edu%2Fdata%2Fhousing-activity%2F%23!%2Factivity%2FMSA%2FHouston-The_Woodlands-Sugar_Land&data=05%7C01%7Cnarayans19%40ecu.edu%7C7f5cb90765ec47a7aa2408dac75aefab%7C17143cbb385c4c45a36ac65b72e3eae8%7C0%7C0%7C638041490631753254%7CUnknown%7CTWFpbGZsb3d8eyJWIjoiMC4wLjAwMDAiLCJQIjoiV2luMzIiLCJBTiI6Ik1haWwiLCJXVCI6Mn0%3D%7C3000%7C%7C%7C&sdata=iVOhBjQdMPU%2FYoRcTc1b4fdbRTRGi4zJz3QnmduQsB0%3D&reserved=0) (2022).
20. Texas Real Estate Research Center, Texas A&M University. Rural Land Prices for Houston (LMA 28). At [https://www.recenter.tamu.edu/data/rural-land/#!/state/Texas/lma/Houston_(LMA_28)](https://nam02.safelinks.protection.outlook.com/?url=https%3A%2F%2Fwww.recenter.tamu.edu%2Fdata%2Frural-land%2F%23!%2Fstate%2FTexas%2Flma%2FHouston_(LMA_28)&data=05%7C01%7Cnarayans19%40ecu.edu%7C7f5cb90765ec47a7aa2408dac75aefab%7C17143cbb385c4c45a36ac65b72e3eae8%7C0%7C0%7C638041490631753254%7CUnknown%7CTWFpbGZsb3d8eyJWIjoiMC4wLjAwMDAiLCJQIjoiV2luMzIiLCJBTiI6Ik1haWwiLCJXVCI6Mn0%3D%7C3000%7C%7C%7C&sdata=upKHQeJ7coZBD4Ybv1LbjX7yItk5H%2BQCnkUxc0c5Dcs%3D&reserved=0) (2021).
21. Huizinga, J., De Moel, H. & Szewczyk, W. Global flood depth-damage functions: Methodology and the database with guidelines (No. JRC105688). Joint Research Centre (Seville site) (2017).
